# Phylogeography, genetic diversity, and connectivity of brown bear populations in Central Asia

**DOI:** 10.1371/journal.pone.0220746

**Published:** 2019-08-13

**Authors:** Odbayar Tumendemberel, Andreas Zedrosser, Michael F. Proctor, Harry V. Reynolds, Jennifer R. Adams, Jack M. Sullivan, Sarah J. Jacobs, Tumennasan Khorloojav, Tuya Tserenbataa, Mijiddorj Batmunkh, Jon E. Swenson, Lisette P. Waits

**Affiliations:** 1 Department of Natural Science and Environmental Health, University of South-Eastern Norway, Bø i Telemark, Norway; 2 Birchdale Ecological, Kaslo, Canada; 3 Gobi Bear Fund Inc, Fairbanks, Alaska, United States of America; 4 Department of Fish and Wildlife Sciences, University of Idaho, Moscow, Idaho, United States of America; 5 Department of Biological Sciences, University of Idaho, Moscow, Idaho, United States of America; 6 Genetics Laboratory, Institute of General and Experimental Biology, Mongolian Academy of Sciences, Ulaanbaatar, Mongolia; 7 Sunshine Village Complex, Bayanzurkh District, Ulaanbaatar, Mongolia; 8 Mongolian-Chinese Joint Molecular Biology Laboratory, Ulaanbaatar, Mongolia; 9 Faculty of Environmental Sciences and Natural Resource Management, Norwegian University of Life Sciences, Ås, Norway; National Cheng Kung University, TAIWAN

## Abstract

Knowledge of genetic diversity and population structure is critical for conservation and management planning at the population level within a species’ range. Many brown bear populations in Central Asia are small and geographically isolated, yet their phylogeographic relationships, genetic diversity, and contemporary connectivity are poorly understood. To address this knowledge gap, we collected brown bear samples from the Gobi Desert (n = 2360), Altai, Sayan, Khentii, and Ikh Khyangan mountains of Mongolia (n = 79), and Deosai National Park in the Himalayan Mountain Range of Pakistan (n = 5) and generated 927 base pairs of mitochondrial DNA (mtDNA) sequence data and genotypes at 13 nuclear DNA microsatellite loci. We documented high levels of mtDNA and nDNA diversity in the brown bear populations of northern Mongolia (Altai, Sayan, Buteeliin nuruu and Khentii), but substantially lower diversity in brown bear populations in the Gobi Desert and Himalayas of Pakistan. We detected 3 brown bear mtDNA phylogeographic groups among bears of the region, with clade 3a1 in Sayan, Khentii, and Buteeliin nuruu mountains, clade 3b in Altai, Sayan, Buteeliin nuruu, Khentii, and Ikh Khyangan, and clade 6 in Gobi and Pakistan. Our results also clarified the phylogenetic relationships and divergence times with other brown bear mtDNA clades around the world. The nDNA genetic structure analyses revealed distinctiveness of Gobi bears and different population subdivisions compared to mtDNA results. For example, genetic distance for nDNA microsatellite loci between the bears in Gobi and Altai (F_ST_ = 0.147) was less than that of the Gobi and Pakistan (F_ST_ = 0.308) suggesting more recent male-mediated nuclear gene flow between Gobi and Altai than between Gobi and the Pakistan bears. Our results provide valuable information for conservation and management of bears in this understudied region of Central Asia and highlight the need for special protection and additional research on Gobi brown bears.

## Introduction

Understanding the phylogeographic patterns and current genetic connectivity of a species provides a greater understanding of evolutionary history and is important for conservation and management decisions [1]. Natural phylogeographic distribution, as well as genetic patterns of diversity and connectivity, have been shaped over evolutionary time by events such as mountain formation and glaciation [2]. However, anthropogenic effects, such as habitat change and fragmentation, commonly disrupt natural evolutionary processes over a much shorter time scale. Large and long-lived animals with slow life histories typically live at low population densities and require large areas, which makes them especially vulnerable to anthropogenic disruption, affecting genetic diversity and connectivity. Habitat fragmentation leading to isolation in populations and individuals has negative demographic and genetic effects [3]. Endangered and geographically isolated populations may benefit from conservation measures that promote genetic and demographic rescue, but knowledge of a species’ genetic history is required to avoid inbreeding and outbreeding depression [4].

The brown bear (*Ursus arctos*) was historically distributed across most of the Northern Hemisphere, but many populations, particularly in the southern portions of their distribution, are now isolated and of conservation concern [5, 6]. Although the phylogeography of brown bears has been studied extensively in many parts of the world [7–10], very little information is available about the populations in Central Asia, many of which are fragmented into several small populations and listed as critically endangered by the International Union for Conservation of Nature (IUCN) [6]. In addition, the taxonomic status, as well as evolutionary history, of brown bears in Central Asia is still uncertain and contested. Based on morphological differences and geographic locations, brown bears in Central Asia have been classified into several different subspecies. For example, brown bears in the Altai region of Russia have been classified variously as East Siberian brown bears (*U*. *a*. *collaris*) [11] or as Mongolian brown bears (*U*. *a*. *jeniseensis*) [12]. Brown bears from the Sayan and Khentii mountains on the Mongolian Plateau have been variously classified as Mongolian [12, 13] or Eurasian brown bears (*U*. *a*. *arctos*) [14, 15]. The question of subspecies classification has direct implications for conservation planning, as it may affect the recognition of a population as an endangered species or subspecies [16].

Only a few studies have investigated the phylogeny of brown bears in Central Asia [17–19]. These mitochondrial DNA (mtDNA)-based molecular studies have identified 3 divergent lineages in Central Asia: clade 3a1, found throughout Eurasia and the Russian Far East; clade 5 on the Tibetan Plateau; and clade 6, found in the Gobi Desert and the Tian Shan and Himalaya mountains [18, 19]. These studies were all based on small sample sizes and phylogenetic relationships were only inferred from the maternally inherited mtDNA. Incorporating nuclear DNA data would lead to a better understanding of phylogenetic relationships of brown bears in the Northern Hemisphere.

In brown bears, as in most mammals, males are generally the dispersing sex and they can disperse over very large distances, whereas females generally have short dispersal distances and often overlap with their maternal home range [20]. In this study, we aimed to evaluate the phylogeographic relationships of the fragmented brown bear populations in Central Asia and provide information crucial for their conservation. We aimed to answer 3 main questions for 6 geographically separate brown bear sampling areas in Central Asia using both maternal mtDNA and bi-parental microsatellite variation. Our questions were: 1) How many mtDNA phylogeographic brown bear groups are present in Central Asia and how do they relate to other phylogeographic bear groups worldwide ? 2) How many nDNA genetic bear groups are present in Central Asia and what is the degree of connectivity? 3) What are the levels of mtDNA and nDNA diversity in bear groups in Central Asia?

## Materials and methods

### Study area and sample collection

**Fig 1:**
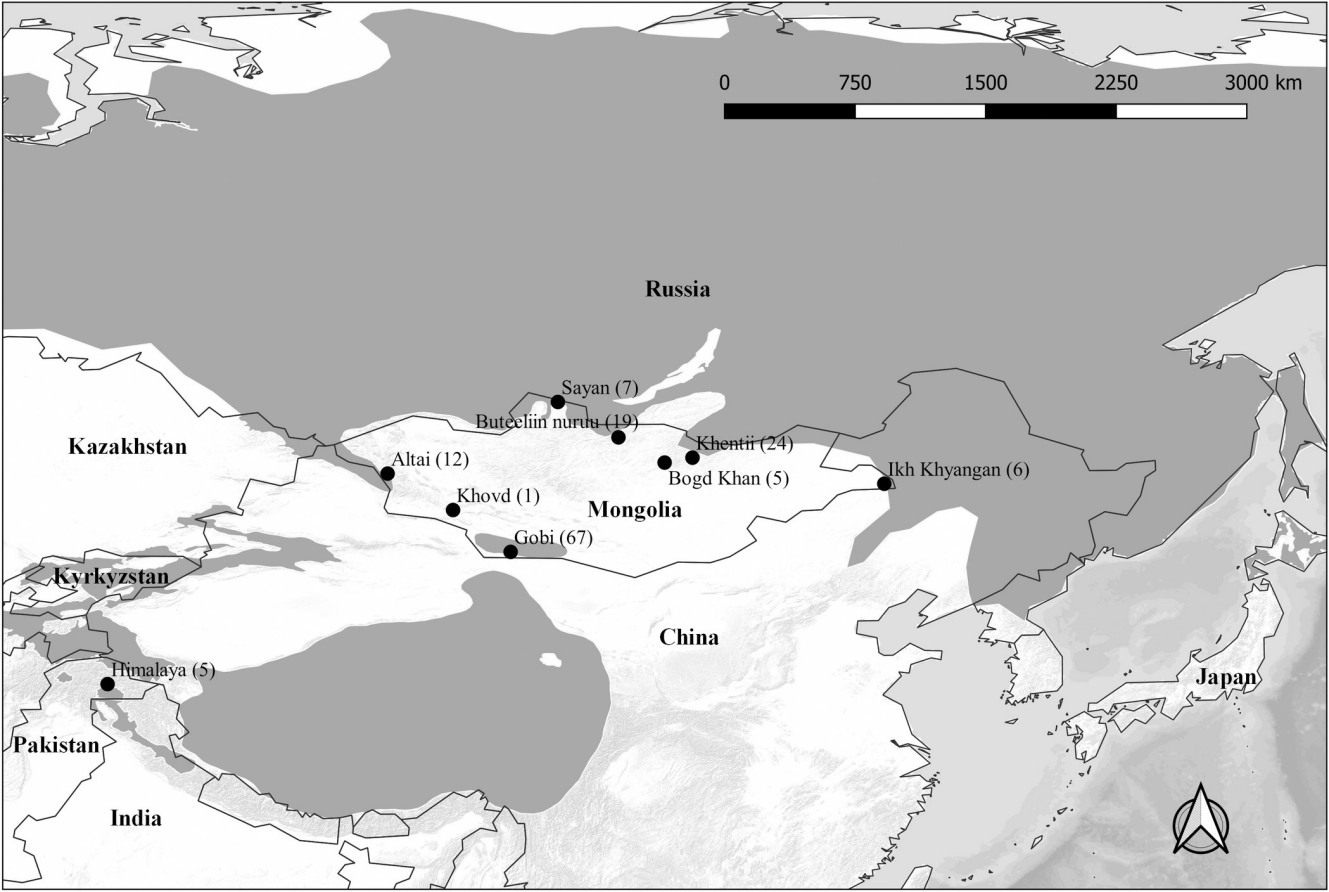
Locations of sampled brown bear populations in Central Asia. Sample sizes are given in parenthesis. Gray shading represents the estimated current brown bear distribution [6].

We collected brown bear samples from 8 geographic locations, including the Great Gobi “A” Strictly Protected Area (hereinafter Gobi) (~43°40´N; ~97°20´E), the Altai (~47°52´N; ~90°88´E), Sayan (~51°20´N; ~101°15´E), Buteeliin nuruu (~49°59´N; ~104°07´E), Khentii (~48°43´N; ~108°30´E), Bogd Khan (~47°40´N; ~107°10´E), and Ikh Khyangan mountains (~46°90´N; ~119°20´E) in Mongolia, and Deosai National Park in Pakistan (hereafter Himalaya) (~35°50´N; ~75°20´E) (Fig 1). We collected 2,360 hair samples using barbed wire hair traps at 14 bait sites in Gobi between 1999 and 2017. Fourteen hair samples, 1 scat, and 65 tissue samples (0.5–1 cm^2^ skin) were collected from local museums, field biologists, and locals from the Khovd (n = 1), Altai (n = 12), Sayan (n = 7), Khentii (n = 29), Buteeliin nuruu (n = 19) and Ikh Khyangan mountains (n = 6) between June 2005 and December 2016. The 5 hair samples from the Himalaya were collected in 1996 [18]. All tissue and skin samples were stored in paper envelopes at room temperature prior to analysis. The permission for sample collection in each area was issued by Mongolian Ministry of Environment and Tourism (formerly the Mongolian Ministry of Nature and Green Development) and Institute of General and Experimental Biology, Mongolian Academy of Sciences. We obtained the necessary permits for import and export of all bear samples from the Convention on International Trade in Endangered Species of Wild Fauna and Flora (CITES) (export: MN1000500, 18/1148; import 12US807212/9 and 18US59229C/9).

### DNA extraction, microsatellite genotyping and analysis

Total genomic DNA from scats, hair, and skin was extracted using the QIAamp DNA Stool kit and Qiagen DNeasy tissue kit (Qiagen Inc., Valencia, CA, USA). We extracted DNA from hair in a room dedicated to low-quality and -quantity genetic samples and included 5–10 guard hair roots in each extraction, if available. We extracted DNA from scat in a biosafety hood in a room dedicated to low-quality and- quantity genetic samples to avoid any cross-contaminations. A negative control was included to test for contamination in each batch of extractions.

Extracted DNA was first screened with 3 mitochondrial DNA species ID primers (H16145, SIDL, and H3R) to ensure the sample was from a bear and to evaluate the sample quality [21]. We chose 13 sufficiently polymorphic microsatellite loci for Gobi bears, including G10B, G10D, G10L, G10M, G10P, G10U, Mu50, and Mu59 [22], D1a [23], REN145P07 [24], Mu51, Mu23, and MU11 [25]. We also included the sex marker, SE47-48 [26]. We used 2 multiplexes including 8 microsatellite primers in the first set and 6 microsatellite primers for the second set for the optimization. All PCR reactions contained approximately 2μl genomic DNA and 5μl of PCR mix with a final concentration of 0.5–0.16 μM of each primer, 1xQIAGEN master mix, 0.5xQ solution. The hair samples were amplified 3–8 times, and samples with better quality DNA, e.g. skin samples, were amplified 2–4 times until a consensus genotype could be determined. For a heterozygous consensus genotype, we required that each allele was observed in at least 2 independent PCRs and, for a homozygous consensus genotype, we required 3 independent PCRs with homozygous results. A negative control was included in all PCRs to test for contamination. We separated the PCR products by size using an Applied Biosystems 3130xl sequencer and scored them using the associated GeneMapper 3.7 software (Applied Biosystems, Foster City, CA, USA).

Based on the probability of identity among siblings statistic P_(ID)_sibs [27], we determined that 10 loci were necessary to achieve < 0.01 probability of 2 siblings having identical genotypes. Consensus genotypes including > 10 loci from all samples were compared using Genalex 6.5 [28] to match samples and distinguish unique individuals. To avoid overestimation and account for undetected genotyping errors, we lumped samples if a mismatch could have been due to allelic dropout [29]. Reliotype [30] was used to test the accuracy of unique genotypes detected in only 1 sample (i.e. single detections) to ensure genotypes met a 95% accuracy threshold.

The genetic diversity within each population was calculated using Arlequin [31] and Microsatellite Analyzer (MSA) [32]. Because our sample sizes from each population were uneven, we calculated allelic richness using ADZE-1.0 [33] with a parameter set to 10 maximum standardized to the sample size (n = 5). We calculated genetic differentiation between populations using pairwise F_ST_ [34] and G”st [35] and between individuals using Principal Coordinate Analysis (PCoA) with Genalex v6.5 [28]. To further identify genetic structure and clustering between geographically isolated populations, we randomly subsampled 27 of 68 identified individuals from Gobi to obtain proportional representation [36] from each identified group before we ran the cluster analysis, STRUCTURE v2.3.4 [37]. We applied a 1,000,000 Markov Chain Monte Carlo sampling scheme, considering samples occurring after the burn-in period of 100,000 samples, and applied an admixture model allowing for correlated allele frequencies between populations. We considered values of k between 1 and 10, repeating each analysis 10 times at each value of k. The most probable clustering was calculated via evaluating the likelihood curves and checking the distribution of Delta K [38] using STRUCTURE HARVESTER v.0.68 [39].

### Mitochondrial DNA sequencing and analysis

We investigated the phylogenetic relationships between brown bear populations in Central Asia using 2 segments of mitochondrial DNA; the cytochrome oxidase-2 (COXII) gene (671bp) and control region (CR) (256 bp). A partial COXII gene was amplified with primers BCOX2F 5'-CTTTGTCAGGGTTAAATTATAGGT-3' and BCOX2R 5'-GGAGAAGTCTGCATTCTCAGT-3' [40]. The CR was amplified in 2 segments. For the first segment, we used (H) 5'-CCTAAGACTAAGGAAGAAG3' and (L)5'-CTTATATGCATGGGGGCACG-3' and, for the second segment, we used the primer sequences (H)5'-CATCGCAGTATGTCCTCG-3' and (L)5'-TACTCGCAAGGATTGCTGG-3' [8]. All PCR reactions were executed using a total volume of 10 μl; including 2μl genomic DNA, 0.5 μM of each primer, 0.2 mM dNTPs, 1.5 mM MgCl, and 0.1 units of *Taq* DNA polymerase. The PCR thermal cycle profiles were as follows: an initial-denaturing step of 94°C for 10 minutes; denaturing step of 95°C for 30 seconds, annealing at 55°C for 30 seconds, and extension at 72°C for 1 minute (35–54 cycles); plus a final extension at 72°C for 10 minutes. The PCR products were stored at 4°C. To remove primers and unincorporated nucleotides, amplified products were purified using the QIAquick PCR purification kit (Qiagen Inc., Valencia, CA, USA). Contaminants were checked during DNA extraction, PCR, and sequencing processes using negative controls (one negative control for each 15–20 samples). The laboratory processes in DNA extraction, PCR, and sequencing were performed in separate rooms to avoid the cross-contamination. Sequences were determined with an Applied Biosystems 3130xl sequencer (Applied Biosystems, Foster City, CA, USA). Sequences were edited by hand using Sequencher version 5.3 (GeneCodes, Ann Arbor, MI, USA) and aligned using MAFFT.v7 [41]. The number of haplotypes, number of polymorphic sites, and haplotype diversity were calculated for the CR and COXII using DNAsp 6 version [42]. The hypervariable sites in the CR sequence were excluded from phylogenetic analyses. Molecular variance (AMOVA) within and among populations was calculated with Arlequin 3.5.2.2 [31]. Median joining analysis was performed in Network 5.0.0.1 (http://www.fluxus-engineering.com) using sequences collected during this study as well as Genbank data. Most samples from Genbank were from extant brown bear populations but also included 3 cave bear samples and 6 fossil brown bear samples (S1 Table).

Due to the extensive amount of research on bear phylogeny based on mtDNA, an additional 115 brown bear haplotypes from populations in mainland Russia, the Middle East, and from the hypothesized sister species, the extinct cave bear (*Ursus spelaeus*, FN390870) [43] and the extant sloth bear (*Melursus ursinus*, EF196662), were retrieved from Genbank for phylogenetic analysis. Sequence availability was greater in the control region (115 CR sequences in total), compared to the COXII region, which had fewer available samples (62 sequences in total). We performed independent analyses of both datasets, as well as a fully concatenated matrix of CR and COXII gene sequences, with all available sequence data. Because many samples had identical sequences (i.e., no substitutions at any site), we pruned all datasets by randomly removing duplicate samples from the same geographic region. This allowed us to reduce the number of identical haplotypes, while retaining geographic representation. Sequences were aligned separately using Muscle v.3.8.31 [44]. Models of molecular evolution for nucleotide alignments were estimated using the AutoModel command in PAUP* v.4.0a [45], where predefined data blocks corresponded to the CR and the COXII genes. The Akaike Information Criterion (AIC), as implemented in PAUP*, was used to identify the highest-ranking models of molecular evolution. All downstream phylogenetic analyses used these partitioning schemes and models.

To infer evolutionary relationships, we performed a phylogenetic analysis using both maximum likelihood and Bayesian criteria. Maximum likelihood trees were estimated using Garli [46]. Each analysis consisted of 25 search replicates and subsequent log files were examined to ensure that each resulted in similar trees and log-likelihood scores, thus indicating that the separate searches had recovered similar topologies. A bootstrap run of 1,000 replicates was performed to assess nodal support. The *sumtrees* function of the dendropy package v.4.0 [47] was used to summarize bootstrap runs on the best tree. Bayesian phylogenetic analyses were conducted in MrBayes v.3.2.1 [48]. Each analysis consisted of 4 Markov chains (using default-heating schemes), sampled every 1,000 generations for a total of 2,000,000 generations. To avoid false stationarity at local optima, we conducted 4 independent runs of each analysis. The stationarity of chains and convergence of parameter estimates was determined by plotting the likelihood scores and other parameter values against generation time using the program Tracer v.1.5 [49]. Stationarity was assumed when all parameter estimates and the likelihood had stabilized. Burn-in was visually assessed and a conservative 25% of trees were discarded. The remaining trees and their associated values were summarized using the *sump* and *sumt* commands in MrBayes. A majority-rule consensus tree showing all compatible partitions from the resulting posterior distribution of topologies was used to recover the posterior probabilities of nodes. Each analysis described above was performed independently on both the full and pruned datasets of the CR and the COXII genes, as well as the full and pruned concatenated dataset of all CR and COXII sequences.

We estimated divergence times in BEAST v.2.5.1 [50], utilizing the full concatenated dataset, as well as the partitioning schemes and nucleotide substitution models described above. We employed a relaxed, lognormal molecular clock to estimate rates of evolution on each lineage and we applied fossil calibrations to 2 nodes, the crown node of the polar bear (*U*. *maritimus*) + brown bear lineage, and the node corresponding to our entire ingroup + sloth bear, to estimate times of divergence across the tree. Calibration priors were centered around the median age reported from previous studies of the fossil record (4.3–6 Ma, placed at the crown node of all *Ursinae* [51]), or derived from ages recovered in other studies that have estimated divergence times using different types of data (0.48–1.1 Ma for the crown polar bear + brown bear node [52–54]). For each node, we applied a normal distribution with minimum and maximum ages reported in the literature falling within the 2^nd^ and 3^rd^ quantiles of the distribution. Five independent analyses were conducted for 200,000,000 generations each, sampling every 10,000 generations, to confirm that independent analyses converged on similar topologies. In addition, we performed an analysis without data to assess the influence of the priors on posterior parameter estimates. Convergence and chain stationarity were assessed in the same way as other analyses in MrBayes. Burn-in was estimated from each trace file separately, burn-in trees were discarded, and then all analyses were combined using LogCombiner [50] and a maximum clade credibility tree was summarized with TreeAnnotator [50].

## Results

We obtained 773 consensus genotypes (≥10 loci) and identified 138 individual bears, including 68 from Gobi (including 1 sample from Khovd), 23 from Khentii, 15 from Buteeliin nuruu, 7 from Sayan, 5 from Bogd Khan, 10 from Altai, 5 from Ikh Khyangan, and 5 from Himalaya using 13 microsatellite loci (Fig 1).

For genetic distance and genetic diversity calculations, individuals from Khentii, Buteeliin nuruu, and Bogd Khan were included as 1 population, due to the short geographic distance between the areas (50–200 km) and results from PCoA and Bayesian clustering analyses. Subsequently, we successfully sequenced 79 samples for the mtDNA CR fragment (256 bp) and 65 samples for the mtDNA COXII fragment (671 bp) (S2 Table).

### Nuclear genetic diversity

All microsatellite loci were polymorphic (2–13 alleles per locus) in 7 of 8 brown bear populations, except 3 microsatellite loci that were monomorphic in the Himalayan sample (n = 5). A total of 85 alleles were generated by 13 microsatellite loci with a range of 77–246 bp. Genetic diversity levels were highly variable among populations in Central Asia, ranging from the lowest values in the Gobi (Ar = 2.07; H_E_ = 0.48) and the Himalaya (Ar = 2.08; H_E_ = 0.38) to the highest values in Khentii+Buteeliin nuruu+Bogd Khan (Ar = 2.98; H_E_ = 0.81) and Altai (Ar = 3.06; H_E_ = 0.78) and Khyangan (Ar = 2.84; H_E_ = 0.72) (Table 1).

**Table 1:** Genetic diversity of brown bear populations in Central Asia based on 13 microsatellite loci.

Populations	n	TNA	Ar	Ho	He
Gobi Desert (GGSPA Mongolia)	68	46	2.07	0.51 (0.19)	0.48 (0.17)
Khentii (Khentii, Buteeliin nuruu and Bogd Khan)	48	101	2.98	0.75 (0.12)	0.81 (0.04)
Sayan (Khuvsgul)	7	82	3.38	0.85 (0.19)	0.85 (0.07)
Altai (Bayan-Ulgii)	12	74	3.06	0.74 (0.14)	0.78 (0.09)
Khyangan (Dornod)	6	82	2.84	0.51 (0.23)	0.72 (0.16)
Himalaya (Pakistan)	5	31	2.08	0.57 (0.39)	0.55 (0.17)
Total	146	127	4.61	0.65	0.74

n, number of individuals; TNA, total number of alleles; Ar, allelic richness per locus calculated for a population based on minimum sample size of 5 diploid individuals; Ho/He, observed/expected heterozygosity; Standard deviations are given in brackets.

### Mitochondrial genetic diversity

The brown bear populations in Central Asia showed a comparatively high genetic diversity, based on CR (π = 0.03 and Hd = 0.89) and COXII (π = 0.01 and Hd = 0.87), especially the populations in the Altai, Khentii+Buteeliin nuruu+Bogd Khan, and Ikh Khyangan mountains (S3 Table and S4 Table). However, the genetic diversity in Gobi and Himalaya was low (Hd = 0.14–0.40), having only 2–3 different haplotypes within each population. We found 25 haplotypes from the 256bp CR fragment, based on 25 variable sites from sequences from 79 individuals, as well as 13 haplotypes from 671bp COXII fragment, with 24 variable sites from 65 sequences, excluding gaps.

Based on CR data, we identified 22 previously undocumented haplotypes. Three haplotypes were reported previously as Gobi-I: Genbank accession number AB010728 from GGSPA [17]; PA136: DQ914409 from Pakistan [8]; and KD1 and AB041258 from Kodiak Island [55] (S2 Table). We detected 12 unique new haplotypes in the COXII datasets, but 1 haplotype (4Sel) from Buteeliin nuruu had the same sequence as 16 brown bear sequences in Genbank: KY419692-4, KY419687, KY419674, KY419654, KY419646-51, KY419639-40 [56] from Perm, Magadan, Krasnoyarsk, and AP012579 [9] from Sakhalin in Russia (S2 Table).

### Genetic structure, differentiation, and phylogeny

#### Mitochondrial DNA level

**Fig 2:**
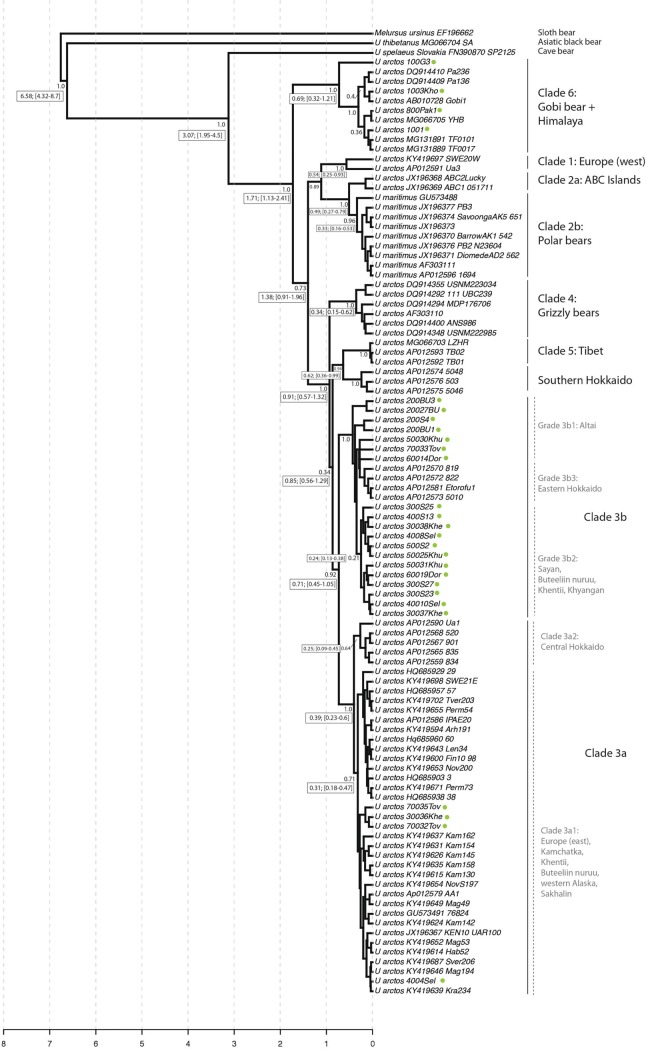
Bayesian phylogenetic tree of brown bears based on mtDNA data (927 bp). The numbers on the nodes are the posterior probability. For simplicity, support values are provided primarily for nodes of interest in the discussion. Divergence time estimates (median divergence estimate, followed by range of estimated times corresponding to the 95% highest posterior density interval (HPD)) are provided in boxes next to their corresponding nodes. For simplicity, we show ages only for those clades discussed in text. Green circles following taxon names correspond to individuals sequenced during the course of this study.

Phylogenetic inference identified 6 primary clades of bears, with varying levels of support, that corresponded to hypothesized lineages from previous work (i.e. Clades 1, 2, 3a, 3b, 4, 5, and 6 [57–60] (Fig 2 and S1 Fig).

Bayesian and maximum likelihood inferences, as well as the topology inferred during the divergence time analysis, were largely congruent with respect to the membership of these primary clades. For simplicity, here we show the maximum clade credibility (MCC) tree, summarized during the divergence dating analysis. This topology was inferred from the concatenated dataset (927 bp) and provides divergence times summarized at nodes of interest. Phylogenetic inference of the full dataset (both Maximum Likelihood and Bayesian topologies) is provided in the supplemental material (S1 Fig).

The brown bear sequences from Central Asia were clustered in clades 3a, 3b, and 6 (Fig 2 and S1 Fig). The bears from Buteeliin nuruu and Khentii mountains had haplotypes in both clades 3a (i.e. the clade found in Europe, the Russian Far East, and Central Hokkaido) and 3b (i.e. the clade found in Eastern Hokkaido). Altai and Ikh Khyangan bears all belonged to Clade 3b. The phylogenetic trees showed that clade 3b had 3 diverging groups, including a grade of lineages, 3b1 in Altai, subclade 3b2 in the Sayan, Khentii, and Ikh Khyangan mountains, and subclade 3b3 in Hokkaido (Fig 2). Based on Maximum Likelihood and Bayesian phylogenetic trees, Gobi and Himalaya bears belonged to clade 6, representing a sister lineage to other extant brown bear populations. However, Gobi and Himalaya bears did not share any haplotypes; haplotypes found in Himalaya diverged from those in the Gobi by 4 to 7 base pairs.

**Fig 3:**
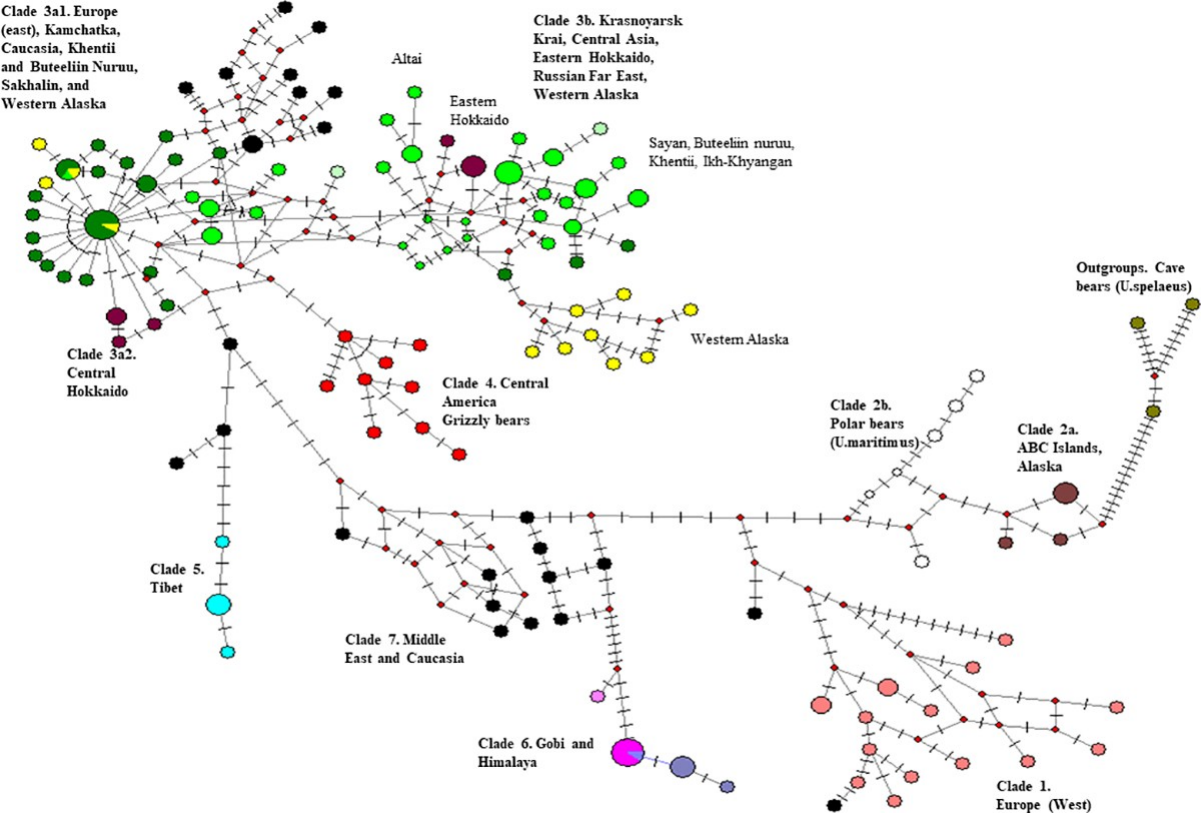
Median-joining haplotype network from brown bear populations around the world. The network is based on mtDNA control region fragment (256 bp). Colors with the nodes indicate the sampling locations; larger ovals identify the clades based on the Bayesian phylogenetic tree. The hatches are the mutation points in the sequences. The small red circles are unobserved haplotypes.

The median-joining haplotype network also showed 3 divergent groups in Central Asia (Fig 3 and S2 Fig). For clade 3a, we found 3 CR haplotypes (CR_Hap8: 4Sel, 6Sel, CR_Hap16: Se5, COII_Hap5) and 2 COXII haplotypes from Khentii and Buteeliin nuruu mountains, which are widely distributed throughout the regions of Perm, Magadan, Krasnoyarsk, Sakhalin in Russia, and western Alaska, USA. The number of mutational steps between 3a and 3b haplotypes in Central Asian populations, where both clades were present, were fewer than between Eurasian and Hokkaido populations, in which only 3a or 3b was present. The haplotypes (300S25, 30036Khe and 4004Sel) in Khentii and Buteeliin mountains were placed as the central haplotypes at the star-shaped network of the 3a and 3b clades.

Based on the divergence time estimate, the clade 3b2 bears in the Sayan-Khentii-Ikh Khyangan-Russian Siberia and clade 3b3 bears in eastern Hokkaido diverged around 0.24 mya (0.13–0.38 kya) from clade 3b1 in Altai. The Maximum Likelihood and Bayesian phylogenetic tree, along with the divergence estimate, indicated that the Himalayan brown bears diverged from Gobi bears 0.69 mya (0.32–1.21 mya) years ago (Fig 2).

#### Nuclear DNA genetic structure

The genetic distance between Gobi and Himalayan brown bears was greater (F_ST_ = 0.339, G''_ST_ = 0.282) than all other pairwise comparisons. Overall, brown bears in both the Gobi and Himalaya had higher pairwise differentiation values (F_ST_ = 0.14–0.22, G_ST_'' = 0.12–0.18) with the Altai, Sayan, and Khentii (included Buteeliin nuruu and Bogd Khan) mountains than these 3 areas had among themselves (F_ST_ = 0.002–0.05; G''_ST_ = 0.02–0.09) (Table 2).

**Table 2:** Comparison of pairwise F_ST_ and G”st among 5 brown bear populations.

		G”ST					
	Population	Altai (n = 11)	Sayan (n = 7)	Khentii (n = 43)	Ikh Khyangan (n = 4)	Himalaya (n = 5)	Gobi (n = 68)
F_ST_	Altai (n = 11)		0.030*	0.038*	0.032*	0.182*	0.133*
	Sayan (n = 7)	0.062*		0.004	-0.002	0.147*	0.120*
	Khentii (n = 43)	0.054*	0.026		0.009	0.162*	0.132*
	Ikh Khyangan (n = 4)	0.086*	0.059	0.052		0.145*	0.143*
	Himalaya (n = 5)	0.222*	0.193*	0.193*	0.218*		0.282*
	Gobi (n = 68)	0.147*	0.139*	0.137*	0.182*	0.308*	

The results are based on nuclear DNA 13 loci microsatellites. The samples size is indicated in parentheses. G”_ST_ and F_ST_ are listed above and below the diagonal, respectively.

*Statistical significance (P < 0.05).

Additionally, pairwise genetic distances between Himalaya and Altai, Sayan, Khentii were greater (F_ST_ = 0.26–0.330, G_ST_'' = 0.14–0.18) than those between Gobi and Altai, Sayan, Khentii (F_ST_ = 0.14–0.21, G''_ST_ = 0.12–0.14) (Table 2). The bears in the Altai, Sayan, and Khentii mountains were sampled from what is likely a continuously occupied range, whereas the Gobi and Himalaya populations are discontinuous in relation to each other and the three northern sampling areas (Fig 1).

**Fig 4:**
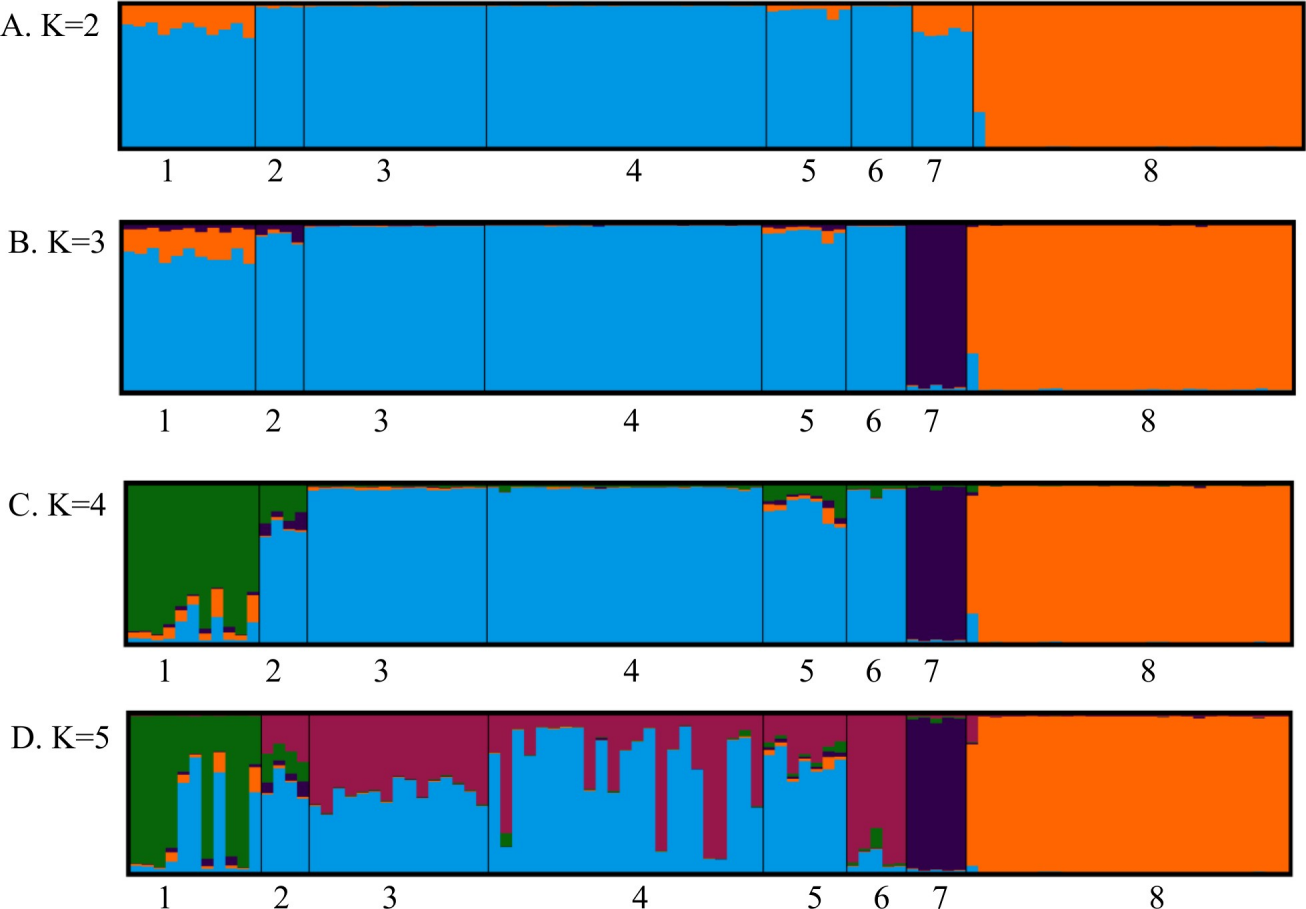
Ancestry plots from the Bayesian clustering (STRUCTURE) from 97 bears sampled in Central Asia. The results are based on 13 nuclear DNA microsatellite loci. Using the Evanno method (S3 Fig), STRUCTURE analysis indicated that the most basic level of subdivision is at K = 2 (S3 Fig), dividing the Gobi brown bears (8, orange) and the rest of the populations in Central Asia (1–7). The numbers correspond to the following geographic areas from Fig 1: 1). Altai; 2). Ikh Khyangan; 3). Buteeliin nuruu; 4). Khentii Mountain; 5). Sayan Mountains; 6). Bogd Khan Mountain adjacent to the Khentii Mountain range in Tov province, Mongolia; 7). Himalaya, Pakistan; 8) Gobi (Great Gobi A Strictly Protected Area, Mongolia).

The Bayesian clustering also strongly supported the split between Gobi and other brown bear populations in the Himalaya, Altai, Sayan, Khentii, Buteeliin nuruu, and Bogd Khan. The population split at K = 2 received the highest likelihood value, based on the Evanno method [38] (S3 Fig) and separated the Gobi bear population from all others. Interestingly, the bear from just north-west of Gobi in Khovd province, which was found dead around at the beginning of the 1900s, had an admixture of ancestry from the bears in the Gobi and Altai Mountain Range. Population splits corresponding to the Himalaya, Altai, and Khentii (including Buteeliin nuruu and Bogd Khan) mountains were highly supported at K = 3–5 respectively (Fig 4).

**Fig 5:**
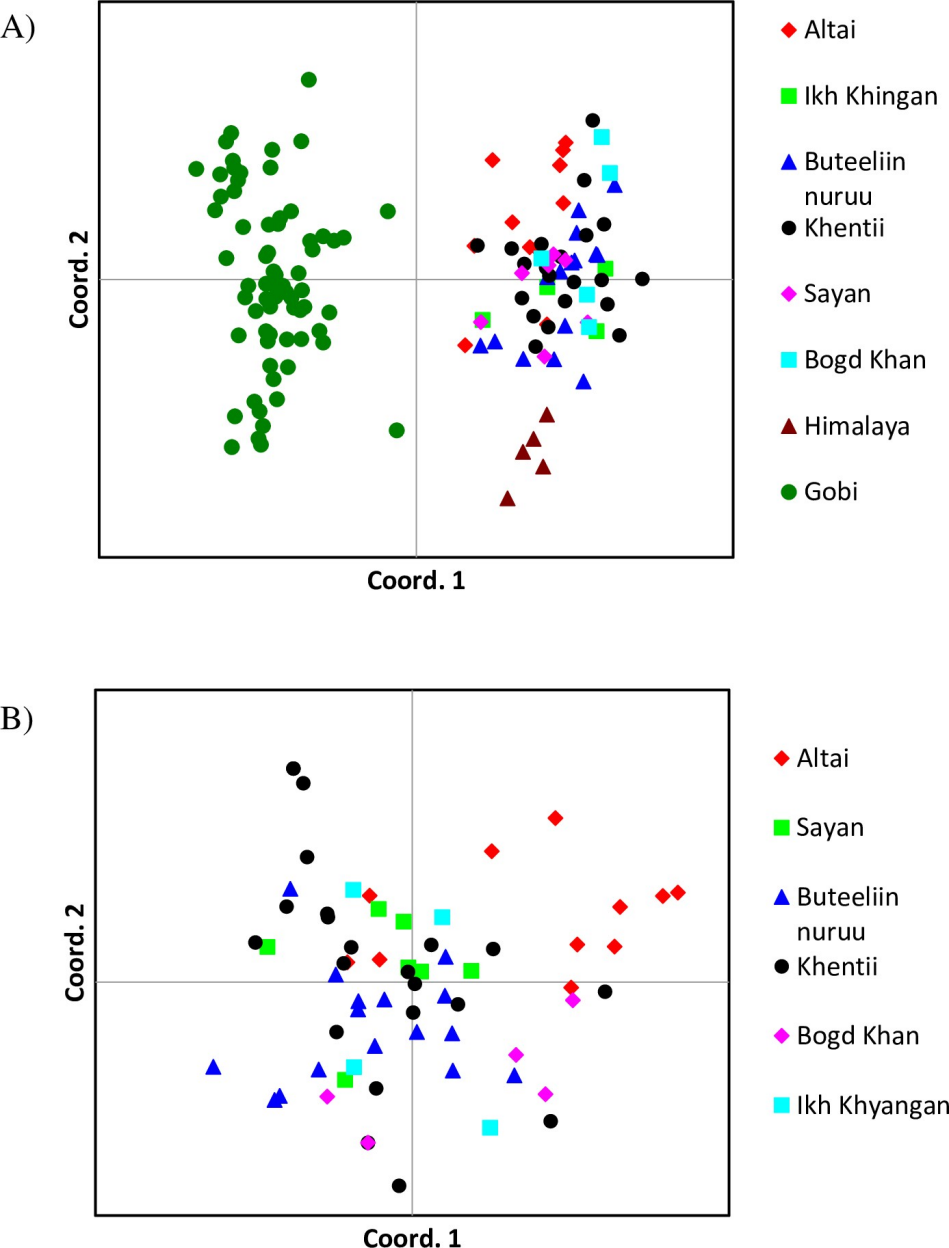
Principal coordinate analysis (PCoA) on individual microsatellite genotypes. A) PCoA based on 13 microsatellite loci from 138 individual brown bears collected at 8 locations across Central Asia. B) PCoA of the bears in 6 geographic locations without Gobi and Himalaya. Each symbol represents a unique individual with symbol color and shape denoting population of origin.

Principal Coordinate Analysis (PCoA) revealed that all Gobi bears clustered together, separately from the rest of the Central Asian brown bears. Himalayan brown bears were distinct from the brown bears in the Altai-Sayan and Khentii mountain ranges (Fig 5A). Therefore, we carried out a second PCoA analysis, excluding individuals from Gobi and Himalaya. The results indicated that bears from the Altai Mountains showed further genetic separation from the remaining populations along both the first and second axes (Fig 5B).

As an additional result, we found an 8 bp deletion in the X chromosome microsatellite locus (SE47-48) in all Gobi bear samples. Also, only a few females (n = 4) from Altai and Ikh Khyangan had allele 232 in 1 of the X chromosomes, but the rest of the sampled brown bear populations had a 240 allele at this locus.

## Discussion

Collecting genetic data and samples from wildlife in remote regions is a difficult task but can provide valuable information about evolutionary history, taxonomy, population connectivity and genetic health of populations. Using mitochondrial and nuclear DNA data collected from previously unsampled brown bear populations in Central Asia, first, we documented high levels of mtDNA and nDNA diversity in the Altai, Sayan, Buteeliin nuruu and Khentii populations, but substantially lower diversity in the brown bear populations in Gobi and Himalaya. Second, our data revealed 3 brown bear mtDNA phylogeographic groups among the bears of the region, with clade 3a1 in Sayan, Khentii, and Buteeliin nuruu mountains, clade 3b in Altai, Sayan, Buteeliin nuruu, Khentii, and Ikh Khyangan, and clade 6 in Gobi and Himalaya. Our results also clarified the phylogenetic relationships with other brown bear mtDNA clades around the world. Third, nDNA clustering analyses revealed different population subdivisions compared to mtDNA results, especially for the brown bear populations in Gobi, Himalaya and Altai.

### Genetic diversity

The highest levels of genetic diversity in both mtDNA and nDNA data were observed in brown bear populations in northern Mongolia. Brown bear populations in Altai, Sayan, Khentii (including Buteeliin nuruu and Bogd Khan), and Ikh Khyangan were hypothesized to be contiguous within Mongolia and with the large bear populations in Russia, China, and Kazakhstan [6, 61]; our results supported that hypothesis. Large population sizes, with high gene flow based on Fst results, likely explains the high mtDNA and nDNA genetic diversity of brown bears in the Altai (Hd = 0.72, Ar = 3.06, H_E_ = 0.78), Sayan (Hd = 0.89, Ar = 3.38, H_E_ = 0.84), Khentii (Hd = 0.77, Ar = 2.98, H_E_ = 0.81), and Ikh Khyangan (Ar = 2.84, H_E_ = 0.72) mountain ranges (Table 1). Because genetic diversity is a relative estimate, depending on the selected loci, direct comparisons with values in the literature are limited. However, genetic diversity of these northern Mongolian populations appears to be comparatively higher than that of the larger brown bear populations that have been assessed with microsatellite loci in North America [62–64] and Europe [65]. Our mtDNA results also revealed the potential historical and contemporary gene flow among bears in these regions (Figs 2 and 3, S1, S2 and S4 Figs), especially between the Sayan, Buteeliiin and Khentii mountain ranges, where individuals are part of the 3b2 clade and populations share common haplotypes CRHap8, CRHap13 (S2 Table and S2 Fig).

The previously undescribed haplotypes found in this study are unique to Central Asian brown bears. Brown bears in the Altai Mountains had 5 novel mtDNA haplotypes (Hap3-Hap7 in CR). Both the Gobi (Hap1_CR and Hap2_CR) and Himalayan populations (Hap26 in CR and Hap13 in COXII) had 2 novel haplotypes (S2 Table), which were previously reported as control region (PA136, PA236) haplotypes [8].

We found that the genetic diversity of both Himalayan and Gobi bears was very low. We chose a set of markers that were polymorphic in Gobi bears to facilitate individual identification of samples collected from hair snares, but 3 of them (G10B, G10L, MU51) were monomorphic for Himalayan bears. Previous studies [66, 67] present more accurate comparisons of genetic diversity of Gobi bears compared to other populations, because they used more microsatellite markers (24 loci) from Gobi and a large sample size of individuals (n = 28) from the Himalaya. Therefore, the previous estimate [67] of overall low diversity (He = 0.29) in Gobi bears, relative to other brown bears around the world, remains the best estimate of genetic diversity of bears in this region. The Gobi bears have clearly been isolated and low in number (< 40) for many decades, which has created significant genetic drift [68].

### Mitochondrial DNA phylogeography

Our analysis of genetic variation of CR and COXII mtDNA sequences revealed that brown bears from Central Asia included 3 divergent haplogroups that clustered into clades 3a1, 3b, and 6. The phylogeographic history of each mitochondrial lineage is examined below.

#### Clade 3a

The brown bears in Clade 3a have been divided into 2 main divergent groups [8, 9]: 1) subclade 3a1 is the most widely distributed clade throughout Eurasia, the Russian Far East and Sakhalin, and western Alaska and has been studied extensively, and 2) subclade 3a2 is only found in Central Hokkaido.

We found several haplotypes on both CR (CR_Hap8, CR_Hap16, CR_Hap19, and CR_Hap25) and the COXII (COII_Hap5, COII_Hap12) in Khentii and Buteeliin nuruu that fit within the 3a1 subclade (S2 Table). Additionally, 3 haplotypes (CR_Hap8, CR_Hap16, COII_Hap5) are also widely distributed throughout Perm, Magadan, Krasnoyarsk, Sakhalin, and western Alaska, suggesting shared historical female ancestry. Several authors [69, 70] have proposed that the Carpathian Mountains was a refuge of ancient 3a1 brown bears, which expanded into Central Europe 54 kya. Other studies have suggested that populations in Asia, especially the Altai-Sayan region, may have been one of the glacial refugia of 3a1 bears during the global cooling period (Kindler et al. 2014) of the late Pleistocene 44–54 kya [56, 71, 72]. We found 1 individual from the Sayan Mountain Range (500Br45Khu) with a private haplotype (CR_Hap19); the rest of the 3a1 bears (n = 13) came from Buteeliin nuruu and Khentii (CR_Hap8, CR_Hap16, CR_Hap25, and COII_Hap4), which are only 100–200 kilometers from the Sayan Mountain Range. Our likelihood inference placed brown bears from Bulgaria and Romania as a sister group to the rest of the 3a1 clade. This relationship is consistent with the results of [56]. Keis et al. (2013) suggested that the recent divergence of subhaplogroups of 3a1 bears in the Khentii Mountains was the result of the last glaciation, as well as postglacial conditions in Eurasia [10]. We recovered the same haplotype (400Sel) in Buteeliin nuruu as has been found in Kodiak Island bears in Alaska (AB041258). This supports the hypothesis of brown bear dispersal from Eurasia to western Alaska after the Bering Land Bridge [73] formed about 11–13 kya, suggesting that 3a1 bears dispersed into western Alaska through Beringia and also migrated into Kamchatka [56].

Our results were consistent with the previous divergence time estimates using the complete mitochondrial genome, i.e., that subclades 3a1 and 3a2 diverged in the Eurasian continent about 53 kya (95 CI: 21–95 kya) and then 3a2 moved earlier than 3a1 to Hokkaido Island through Sakhalin [9, 56].

#### Clade 3b

We found brown bears in clade 3b throughout most sampling locations, including the Altai, Sayan, Buteeliin nuruu, Khentii, and Ikh Khyangan mountains. Previously brown bears of this clade have been found in Eastern Hokkaido, the Russian Far East, eastern Alaska, and northern Canada [9, 17, 58]. The 3 subclades within 3b bears, including 3b1 in Altai, 3b2 in the Sayan, Buteeliin nuruu, Khentii, and Ikh Khingan mountain ranges, and 3b3 in eastern Hokkaido did not have strong statistical support, but they were also identified in our network analysis. A previous study found a few bears with 2 new haplotypes (H_337 and T_12) in southern Tomsk and Krasnoyarsk Krai in Russia [72], which is ~300–400 km from our sampled populations in the Sayan and Khentii mountains. These 2 haplotypes clustered into the subclade 3b2 in our dataset, which also infers historic genetic connectivity between these populations. Based on the high genetic variation in 3b1 bears from the Altai Mountains, as well as the phylogenetic analysis, we suggest that a potential ancestral glacial refuge of clade 3b may have existed in the Altai Mountain Range, from which they perhaps dispersed into central Russia, the Russian Far East, and western Alaska during the late Pleistocene between 0.13–0.38 mya. The environmental conditions in the Altai and Sayan regions (2 geographically close mountain systems) were similar during the Last Glacial Maximum [74].

#### Clade 6. Gobi and Himalayan brown bears

Although these two geographically distant populations are in the same clade, their haplotypes differed by 4 bp in COXII (671bp) in the protein coding region and 1–2 bp in the control region (256 bp). The first phylogenetic paper on Gobi bears [17] found that they were more closely related to European brown bears of the western lineage than Asian bears of the eastern lineage, based on partial CR (269 bp) using UPGMA and neighbor joining tree analyses [75]. That work included only one sample from the Gobi and a few samples from Hokkaido, Tibet, and Europe. Subsequent studies [8, 18] found that Gobi and Himalayan brown bears were closely related and grouped together into clade 6, based on the partial CR fragment. Our Bayesian and Maximum Likelihood analyses, using both mtDNA CR and COXII and with an increased sample size from the Gobi and Deosai National Park, Pakistan, corroborated the results of Miller et al. (2006) and Galbreath et al. (2007) [8, 18]. However, while our phylogenetic analyses are unable to resolve the relationships among Himalayan and Gobi bears, our divergence dating analysis suggests the members of this clade (Himalayan brown bears + Gobi bears) began diverging between 0.32–1.21 mya.

### Nuclear microsatellite DNA genetic groups

Our results based on nDNA analyses showed different patterns in genetic differentiation from the mtDNA results. Pairwise F_ST_ and G”_ST_ between bears from the Gobi and Himalaya (F_ST_ = 0.308, G”_ST_ = 0.282) was greater than between the Gobi and Altai, Sayan, Khentii, and Ikh Khyangan (Fst = 0.137–0.182, G”_ST_ = 0.120–0.143), suggesting more recent male-mediated nuclear gene flow between the Gobi and the northern sampling areas than between the Gobi and the Himalayan bears. Both the STRUCTURE and PCoA analyses indicated that Gobi bears are genetically distinct and divergent from the bears in Himalaya, the Altai-Sayan region, and Khentii (Figs 4 and 5). Small and isolated populations, like Gobi bears [67], are subject to genetic drift, mediating reduction in genetic diversity.

In contrast to the mtDNA (clade 6) results, the genetic distance for nDNA microsatellite loci between the bears in Gobi and the Altai (F_ST_ = 0.147) was closer than that of the Gobi and Deosai National Park in the Himalayan Mountain Range (F_ST_ = 0.308). Bipaternal microsatellite markers reveal more recent gene flow history than mtDNA analyses as well as male-mediated gene flow and may therefore be more relevant for discerning current relationships important for management and conservation. This discordant pattern has also been observed in the ABC Islands in Alaska and in Scandinavia, where male brown bear gene flow has been documented using nuclear microsatellite markers [62, 65]. However, studies based on mtDNA sequences found two distinct and divergent clades between coastal and mainland brown bears in North America and between northern and southern Scandinavia [7, 58, 76].

Understanding both historic and contemporary gene flow among geographically structured populations provides information useful in both a basic evolutionary context and relevant to management and conservation. Male brown bears exhibit great variability in average dispersal distances [77], but occasionally disperse over long distances (467 km [78]). Accordingly, additional studies using nDNA markers (microsatellites or SNPs) showing biparental gene flow are essential to reveal a clearer picture of current conditions, including geographically isolated populations. Although mitochondrial DNA reveals long-term evolutionary history, it provides only half the story, the maternal history of a species [79]. Female brown bears exhibit philopatric behavior [77, 80], which has a big impact on mtDNA patterns [58]. Because of the high rates of change in microsatellite loci, compared to the site-wise mutation rates in noncoding DNA, the mutation rates are greater (0.0001 per locus per generation in general) than the point mutation in mtDNA [81]. Thus, our microsatellite nDNA analysis was important to investigate the more recent genetic relationships of the brown bear populations in Central Asia during the last several hundreds and thousands of years.

Interestingly, the single historical sample (~100 years old) from Khovd showed admixed ancestry in the STRUCTURE and PCoA analyses, which suggests relatively recent gene flow between the bears in the Gobi and Altai. Previously (before 1940), the distribution of Gobi bears was larger than their current distribution [82, 83]. Sign of Gobi bears was found ~70–100 km north of the current Gobi bear distribution at Edren Ridge (~43° 80’N; 97° 20’E) and ~50 km east in the Tost Mountain (43° 12’N; 100° 36’E). However, their distribution declined dramatically during 1940–1970, due to the establishment of human settlements (e.g. Ekhiin gol and Bayantooroi oases) close to water sources [14, 84]. The Trans-Altai Gobi Mountains are considered to be the eastern branch of the Tian Shan Mountains and the eastern spurs of the Mongolian Altai Mountains. Thus, it appears that at least some male gene flow has occurred more recently between the brown bears in Gobi and Altai Mountains than between Gobi and bears in Pakistan, which is consistent with the biogeographic history of this area [85, 86].

STRUCTURE and PCoA analyses indicated that the Gobi brown bear population was the most divergent of all groups sampled. However, there was also additional informative population structure revealed in these analyses. STRUCTURE at K = 3 and the PCoA results revealed that the Himalaya population (mtDNA clade 6a) is the next most divergent followed by the Altai Mountain population (mtDNA, clade 3b1). The historical corridors between those two populations could have been the ridges of the Ugam, Pskem, Kirgiz, Talaz Alatau, Zailiy Alatau, Kungey Alatau, Ter Alatau, Ketmen, Dzhungar Alatau, Saure, Tarbagataye, and Southern Latay regions in Kyrgystan, Kazakstan, China, and the Zhunghar Gobi in southwestern Mongolia [87].

The degree of genetic differentiation observed among the Gobi and Himalayan populations is larger than the average Fst between distinctive subpopulations in northwestern Europe (0.051) [88]. Interestingly, the genetic differentiation between the southern (clade 1) and northern (clade 3a) subpopulations of Scandinavian brown bears has decreased from F_ST_ = 0.051 to F_ST_ = 0.014 during 20 years [89], because of an annual population increase of 4.5% and increased gene flow between these subpopulations [77, 90]. In contrast, a population genetic study [64] found a high pairwise F_ST_ (0.23) between brown bear populations in the South Selkirk Mountains in North America, which has been isolated for several decades from the neighboring populations across a 5-km wide valley filled containing a town and rural farms. Due to this restriction of gene flow for the small population (≤100) in the area [91], genetic diversity (He = 0.54) was reduced, compared to the neighboring populations (He = 0.64–0.68).

For bears in the Sayan, Buteeliin nuruu and Khentii, and Ikh Khyangan mountain ranges, our results indicate substantial gene flow, according to the results in a pairwise F_ST_ values (0.026), STRUCTURE, and PCoA analyses. The Sayan, Buteeliin nuruu, and Khentii bears currently have haplotypes belonging to both clades 3a1 and 3b. Therefore, there is still potential gene flow between the populations in the Altai and Sayan mountains through the Siilkhem Mountains, although our study detected that the bears in the Altai have novel mtDNA haplotypes of the 3b clade only in this region, as well as indications of divergence in both STRUCTURE and PCoA analyses using microsatellite loci. Anthropogenic factors, such as human land use, grazing, farming, and new settlements in Mongolia and Russia, may now restrict movements between populations in Altai and Sayan.

Interestingly, the Altai and Ikh Khyangan populations have genetic similarities, including 3b haplotypes, and small degree of shared ancestry based on STRUCTURE analyses. Also in support of the genetic link between these separated bear populations was a unique sex-specific X chromosome loci (SE47-48) loci, allele 232 that we found in all Gobi bears and in 2 females from each Altai and Ikh Khyangan. These 4 bears were heterozygous containing a 232 allele, as found in the Gobi, and a 240 allele, as found in other brown bear populations. Given that that the geographic distance between the Altai and Ikh Khyangan sampling areas (> 2000 km) is the furthest among the populations we sampled, these genetic similarities may be explained through shared historical ancestry and connectivity. For instance, the coastal bears in Alaska (mtDNA, 3a1 and 3b clades) and Kodiak bears (mtDNA, clade 3a1) which crossed the Bering Strait were eventually cut off with no gene flow, but still shared similar haplotypes with clade 3 bears in Eurasia.

### Future conservation recommendations

Brown bear populations are classified as threatened or endangered across most of Central Asia, due to anthropogenic effects combined with climate change [6, 15]. For effective wildlife conservation and management of endangered populations, it is important to understand population distinctiveness by defining Evolutionary Significant Units (ESU) or Management Units (MU), based on genetic data, ecology, life history, and adaptive differences. Although our mtDNA and nuclear microsatellite DNA results differed, our combined genetics results indicated 3 divergent groups, the Gobi, Himalaya and Altai, which could be potential conservation units. Brown bear populations in the Altai, Sayan, Buteeliin nuruu, Khentii, and Ikh Khyangan have high genetic diversity and are likely connected with the large populations in Russia and China. However, anthropogenic influences, such as hunting pressure, forest fires, and habitat loss due to grazing and development, represent potential threats to these populations. Due to the small population size and isolated status (n = 22–31; [67]) of the Gobi brown bears, this population warrants special protection and additional research. This may include establishing new protected areas to allow natural recolonization of nearby adjacent areas that were recently inhabited by bears. It is also possible that genetic and demographic augmentation from other Central Asian populations may be required to develop these complementary adjacent populations to decrease extirpation risk through development of a metapopulation [92].

## Supporting information

S1 Table Previously published ancient DNA information. Ancient DNA sequences from brown bears and extinct cave bear were retrieved from Genbank database with their corresponding accession number, species name, sample ID, references and sampling location. The age estimates were based on uncalibrated radiocarbon years (years before present, years.B.P.) as well as BEAST posterior estimates (also included 95% highest posterior density interval, HPD).
(DOCX)

S2 Table Brown bear haplotype distribution in Central Asia based on mitochondrial DNA sequence data. The concatenated sequencing dataset includes 671 bp COXII and 261bp Control Region. Twenty-two new haplotypes for control region sequences and 12 new haplotypes from COXII were identified in this study. Concatenated column gives the sample ID used in the phylogenetic and divergence time analyses. The previously reported haplotypes are indicated with the symbol (§) and the Genbank ID is in parentheses in the sample ID column.
(DOCX)

S3 Table Genetic diversity estimates within-populations based on brown bear COXII mitochondrial DNA data (671 bp). Description: n, number of individuals; S, variable sites; Haplotype (gene) diversity, Hd; Nucleotide diversity, π; Average number of nucleotide differences, K.
(DOCX)

S4 Table Genetic diversity within-populations, based on brown bear Control Region mitochondrial DNA data. Description: Gaps are excluded and the total sites 243 bp out of 261 bp. n, number of individuals; S, variable sites; Haplotype (gene) diversity, Hd; Nucleotide diversity, π; Average number of nucleotide differences, K.
(DOCX)

S1 Fig Phylogenetic trees from analysis of brown bears, based on 927 bp mtDNA sequence data. The concatenated sequencing dataset (927 bp) includes 671 bp COXII and 256 bp Control Region. a) Bayesian and b) Likelihood analyses. The samples with green dots are from our study.
(TIF)

S2 Fig Median-joining haplotype network from brown bear populations around the world. The concatenated sequencing dataset (927 bp) includes 671 bp COXII and 256 bp Control Region. Colors in each node indicate the geographic sampling locations; the size of the node indicate frequency of the haplotype. The hatches represent mutational differences in the sequences. The names of the new haplotypes discovered in this study are given in the underlined text on the nodes.
(TIF)

S3 Fig Likelihood curves using the Evanno method [38]. The results are based on 13 loci microsatellite dataset of Asian brown bears. A) The DeltaK results support K = 2 as the most basic level of subdivision (Fig 4), which splits the Gobi bears from the Himalayan-Eurasian Brown bears in the STRUCTURE analysis. B) STRUCTURE likelihood curve for K = 1–8.
(TIF)

S4 Fig Structure results for K = 5, based on 13 brown bear microsatellite loci. Colors and pie charts represent proportions of the ancestry belonging to each K. Pie charts and names (with sample size) are in the general locations. The current brown bear distribution [6] is highlighted as orange.
(TIF)
