# Supplementary material for: Phylogeography, genetic diversity, and connectivity of brown bear populations in Central Asia
Source: PLoS One. 2019 Aug 13;14(8):e0220746. doi: 10.1371/journal.pone.0220746 (PMC6692007; doi:10.1371/journal.pone.0220746)
Supplement: S1 Table — Ancient DNA sequences from brown bears and extinct cave bear were retrieved from Genbank database with their corresponding accession number, species name, sample ID, references and sampling location. The age estimates were based on uncalibrated radiocarbon years (years before present, years.B.P.) as well as BEAST posterior estimates (also included 95% highest posterior density interval, HPD). (DOCX) [file pone.0220746.s001.docx]

**S1 Table. Previously published mtDNA sequence information**

| # | **Accession number** | **Genus** | **Species** | **Sample ID** | **Locality** | **Period** | **C14 Age (YBP)** | **Genome part** | **Sequence length (bp)** | **References** |
| --- | --- | --- | --- | --- | --- | --- | --- | --- | --- | --- |
| 1 | AB010725 | Ursus | arctos | JAPAN-1 | Northern Hokkaido | modern |  | partial D-loop | 268 | [1] |
| 2 | AB010727 | Ursus | arctos | TIBET-1 | Tibet | modern |  | partial D-loop | 269 | [1] |
| 3 | AB010728 | Ursus | arctos | Gobi-1 | GGSPA | modern |  | partial D-loop | 267 | [1] |
| 4 | AB041258 | Ursus | arctos | KD1 | Kodiak | modern |  | partial D-loop | 268 | [2] |
| 5 | AF303110 | Ursus | arctos |  | Canada | modern |  | complete mtdna genome | 17020 | [3] |
| 6 | AF303111 | Ursus | maritimus | Ursus maritimus | Canada | modern |  | complete mtdna genome | 17017 | [3] |
| 7 | AJ809333 | Ursus | arctos | Ramesch 1 | Ramesch cave, Austria | ancient | 47,420 | partial D-loop | 270 | [4] |
| 8 | AJ809334 | Ursus | arctos | Winden 1 | Winden cave, Austria | ancient | 39,940 | partial D-loop | 270 | [4] |
| 9 | AM411397 | Ursus | arctos | AKO1 | Akouker cave, Algeria | ancient | 1679 ± 35 | partial D-loop | 272 | [5] |
| 10 | AM411400 | Ursus | arctos | TAK1 | Takouatz cave, Algeria | ancient | 9620 ± 200 | partial D-loop | 271 | [5] |
| 11 | AP012559 | Ursus | arctos | 834 | Central Hokkaido | modern |  | complete mtdna genome | 16962 | [6] |
| 12 | AP012561 | Ursus | arctos | 2231 | Central Hokkaido | modern |  | complete mtdna genome | 16936 | [6] |
| 13 | AP012564 | Ursus | arctos | 2213 | Central Hokkaido | modern |  | complete mtdna genome | 16944 | [6] |
| 14 | AP012565 | Ursus | arctos | 835 | Central Hokkaido | modern |  | complete mtdna genome | 16943 | [6] |
| 15 | AP012566 | Ursus | arctos | 4293 | Central Hokkaido | modern |  | complete mtdna genome | 16923 | [6] |
| 16 | AP012567 | Ursus | arctos | 901 | Central Hokkaido | modern |  | complete mtdna genome | 16941 | [6] |
| 17 | AP012568 | Ursus | arctos | 520 | Central Hokkaido | modern |  | complete mtdna genome | 16932 | [6] |
| 18 | AP012570 | Ursus | arctos | 819 | Eastern Hokkaido | modern |  | complete mtdna genome | 16896 | [6] |
| 19 | AP012571 | Ursus | arctos | 617 | Eastern Hokkaido | modern |  | complete mtdna genome | 16925 | [6] |
| 20 | AP012572 | Ursus | arctos | 822 | Eastern Hokkaido | modern |  | complete mtdna genome | 16916 | [6] |
| 21 | AP012573 | Ursus | arctos | 5010 | Eastern Hokkaido | modern |  | complete mtdna genome | 16895 | [6] |
| 22 | AP012574 | Ursus | arctos | 5048 | Southern Hokkaido | modern |  | complete mtdna genome | 16967 | [6] |
| 23 | AP012575 | Ursus | arctos | 5046 | Southern Hokkaido | modern |  | complete mtdna genome | 16946 | [6] |
| 24 | AP012576 | Ursus | arctos | 503 | Southern Hokkaido | modern |  | complete mtdna genome | 16943 | [6] |
| 25 | AP012577 | Ursus | arctos | 515 | Southern Hokkaido | modern |  | complete mtdna genome | 16975 | [6] |
| 26 | AP012578 | Ursus | arctos | 518 | Southern Hokkaido | modern |  | complete mtdna genome | 16932 | [6] |
| 27 | AP012579 | Ursus | arctos | AA1 | Sakhalin | modern |  | complete mtdna genome | 16945 | [6] |
| 28 | AP012580 | Ursus | arctos | AA3 | Kunashri | modern |  | complete mtdna genome | 16910 | [6] |
| 29 | AP012581 | Ursus | arctos | Etorofu1 | Etorofu | modern |  | complete mtdna genome | 16953 | [6] |
| 30 | AP012585 | Ursus | arctos | IPAE1 | Ural Mountains | modern |  | complete mtdna genome | 16955 | [6] |
| 31 | AP012586 | Ursus | arctos | IPAE20 | Ural Mountains | modern |  | complete mtdna genome | 16955 | [6] |
| 32 | AP012587 | Ursus | arctos | IPAE50 | Ural Mountains | modern |  | complete mtdna genome | 16962 | [6] |
| 33 | AP012589 | Ursus | arctos | Nov2 | Novgorod | modern |  | complete mtdna genome | 16945 | [6] |
| 34 | AP012590 | Ursus | arctos | Ua1 | Bulgaria, Balkan Mountains | modern |  | complete mtdna genome | 16929 | [6] |
| 35 | AP012591 | Ursus | arctos | Ua3 | Bulgaria, Balkan Mountains | modern |  | complete mtdna genome | 16941 | [6] |
| 36 | AP012592 | Ursus | arctos | TB01 | Tibet | modern |  | complete mtdna genome | 16965 | [6] |
| 37 | AP012593 | Ursus | arctos | TB02 | Tibet | modern |  | complete mtdna genome | 16945 | [6] |
| 38 | AP012596 | Ursus | maritimus | 1694 | Alaska, USA | modern |  | complete mtdna genome | 16971 | [6] |
| 39 | DQ914292 | Ursus | arctos | 111_UBC239 | Central BC, Canada | historical (1939) | | partial D-loop | 229 | [7] |
| 40 | DQ914293 | Ursus | arctos | UBC18 | Central BC, Canada | historical (1935) | | partial D-loop | 229 | [7] |
| 41 | DQ914294 | Ursus | arctos | MDP176706 | North Cont.divide, USA | modern |  | partial D-loop | 229 | [7] |
| 42 | DQ914348 | Ursus | arctos | USNM222985 | Central Colorado, USA | modern |  | partial D-loop | 229 | [7] |
| 43 | DQ914351 | Ursus | arctos | BMP113385 | Central Colorado, USA | historical (1904) | | partial D-loop | 229 | [7] |
| 44 | DQ914355 | Ursus | arctos | USNM223034 | Wasatch Mts, (USA) | historical (1916) | | partial D-loop | 229 | [7] |
| 45 | DQ914362 | Ursus | arctos | USNM213000 | San Juan Mts, (USA) | unknown |  | partial D-loop | 229 | [7] |
| 46 | DQ914400 | Ursus | arctos | ANS986 | Northern Mexico | historical (1861) | | partial D-loop | 229 | [7] |
| 47 | DQ914401 | Ursus | arctos | UBC17156 | Central BC, Canada | modern |  | partial D-loop | 229 | [7] |
| 48 | DQ914406 | Ursus | arctos | R252 | Far East, Russia | modern |  | partial D-loop | 229 | [7] |
| 49 | DQ914407 | Ursus | arctos | 50 | Tibet | modern |  | partial D-loop | 229 | [7] |
| 50 | DQ914408 | Ursus | arctos | 49 | Iran | modern |  | partial D-loop | 229 | [7] |
| 51 | DQ914409 | Ursus | arctos | Pa136 | Pakistan | modern |  | partial D-loop | 229 | [7] |
| 52 | DQ914410 | Ursus | arctos | Pa236 | Pakistan | modern |  | partial D-loop | 229 | [7] |
| 53 | DQ914411 | Ursus | arctos | G106 | Greek zoo | modern |  | partial D-loop | 229 | [7] |
| 54 | EF033706 | Ursus | arctos | UA-8 | ABC1 | modern |  | partial D-loop | 229 | [7] |
| 55 | EF033710 | Ursus | arctos | UA-9 | ABC5 | modern |  | partial D-loop | 229 | [7] |
| 56 | EF033728 | Ursus | maritimus | UA-14 | Norway | modern |  | partial D-loop | 229 | [7] |
| 57 | EF033730 | Ursus | maritimus | UA-16 | West Russia | modern |  | partial D-loop | 229 | [7] |
| 58 | EF033731 | Ursus | arctos | UA-20 | Range1, Alaska, USA | modern |  | partial D-loop | 229 | [7] |
| 59 | EF033733 | Ursus | arctos | UA-26 | Alaska, USA | modern |  | partial D-loop | 229 | [7] |
| 60 | EF033826 | Ursus | arctos | UA-31 | Katmai, Alaska, USA | modern |  | partial D-loop | 229 | [7] |
| 61 | EF033855 | Ursus | arctos | UA-21 | Kluane, Alaska, USA | modern |  | partial D-loop | 229 | [7] |
| 62 | EF033999 | Ursus | arctos | UA-39 | North Cont.divide, USA | modern |  | partial D-loop | 229 | [7] |
| 63 | EF196662 | Melursus | ursinus |  | China | modern |  | complete mtdna genome | 16817 | [8] |
| 64 | EU497665 | Ursus | arctos |  | Guzet shelter, Ariege, France | ancient | unknown | complete mtdna genome | 16753 | [9] |
| 65 | EU526782 | Ursus | arctos | EA18 | Finland | modern |  | partial D-loop | 665 | [10] |
| 66 | EU526792 | Ursus | arctos | EA28 | Finland | modern |  | partial D-loop | 664 | [10] |
| 67 | EU526794 | Ursus | arctos | EB2 | Finland | modern |  | partial D-loop | 664 | [10] |
| 68 | EU526806 | Ursus | arctos | KA4 | Kamchatka | modern |  | partial D-loop | 664 | [10] |
| 69 | EU526807 | Ursus | arctos | KA5 | Kamchatka | modern |  | partial D-loop | 665 | [10] |
| 70 | EU574911 | Ursus | arctos | 61 | Alaska | modern |  | partial cyt b gene and d-loop | 589 | [11] |
| 71 | FN292971 | Ursus | arctos | UAS11 | Lebanon | historical (1870) | | partial D-loop | 267 | [5] |
| 72 | FN292974 | Ursus | arctos | UAS9 | Iran | modern |  | partial D-loop | 271 | [5] |
| 73 | FN292978 | Ursus | arctos | UAS3 | Middle East | modern |  | partial D-loop | 267 | [5] |
| 74 | FN292980 | Ursus | arctos | UAS5 | Middle East | modern |  | partial D-loop | 267 | [5] |
| 75 | FN390870 | Ursus | spelaeus | SP2125 | Medvedia jaskyna, Slovakia | ancient | unknown | complete mtdna genome | 16781 | [12] |
| 76 | GU057343 | Ursus | arctos | MM197805 | Caucasia | modern |  | partial D-loop | 351 | [13] |
| 77 | GU057344 | Ursus | arctos | MM197801 | Caucasia | modern |  | partial D-loop | 351 | [13] |
| 78 | GU057347 | Ursus | arctos | MM197804 | Caucasia | modern |  | partial D-loop | 351 | [13] |
| 79 | GU057353 | Ursus | arctos | MM197829 | Caucasia | modern |  | partial D-loop | 351 | [13] |
| 80 | GU057367 | Ursus | arctos | MM197820 | Caucasia | modern |  | partial D-loop | 351 | [13] |
| 81 | GU573488 | Ursus | maritimus |  | Poolepynten, Svalbard | modern |  | complete mtdna genome | 16898 | [14] |
| 82 | GU573489 | Ursus | arctos | 14KB | Baranof Alaska | modern |  | complete mtdna genome | 16818 | [14] |
| 83 | GU573491 | Ursus | arctos | 76824 | Kodiak Alaska | modern |  | complete mtdna genome | 16893 | [14] |
| 84 | HE657199 | Ursus | arctos | Uar001 | Romania | modern |  | partial D-loop | 682 | [15] |
| 85 | HE657200 | Ursus | arctos | Uar004 | Romania | modern |  | partial D-loop | 682 | [15] |
| 86 | HE657208 | Ursus | arctos | UarBT18 | Norway | modern |  | partial D-loop | 683 | [15] |
| 87 | HE657212 | Ursus | arctos | UarC68 | Sweden | modern |  | partial D-loop | 683 | [15] |
| 88 | HE657214 | Ursus | arctos | UarCar02 | Romania | modern |  | partial D-loop | 682 | [15] |
| 89 | HE657220 | Ursus | maritimus | U.maritimus | Greenland | modern |  | partial D-loop | 682 | [15] |
| 90 | HE657232 | Ursus | maritimus | U.maritimus | Iceland | modern |  | partial D-loop | 682 | [15] |
| 91 | HG426320 | Ursus | arctos | UarABCM21 | ABC mainland | modern |  | partial D-loop | 624 | [16] |
| 92 | HG426328 | Ursus | arctos | UarAKK33 | Alaska | modern |  | partial D-loop | 626 | [16] |
| 93 | HG426335 | Ursus | arctos | UarAKM28 | Alaska Mainland | modern |  | partial D-loop | 625 | [16] |
| 94 | HG426341 | Ursus | arctos | UarBra2 | East Alaska | modern |  | partial D-loop | 625 | [16] |
| 95 | HG426365 | Ursus | arctos | UarKamK20 | Kamchatka | modern |  | partial D-loop | 625 | [16] |
| 96 | HG426376 | Ursus | arctos | UarKar7 |  | modern |  | partial D-loop | 625 | [16] |
| 97 | HG426416 | Ursus | arctos | UmaBBL53 |  | modern |  | partial D-loop | 615 | [16] |
| 98 | HQ602652 | Ursus | arctos | RH0026 | Croatia | modern |  | partial D-loop | 267 | [17] |
| 99 | HQ685903 | Ursus | arctos | 3 | North Estonia | modern |  | complete mtdna genome | 16762 | [18] |
| 100 | HQ685910 | Ursus | arctos | 10 | North Estonia | modern |  | complete mtdna genome | 16771 | [18] |
| 101 | HQ685929 | Ursus | arctos | 29 | North Russia | modern |  | complete mtdna genome | 16771 | [18] |
| 102 | HQ685938 | Ursus | arctos | 38 | East Russia | modern |  | complete mtdna genome | 16780 | [18] |
| 103 | HQ685941 | Ursus | arctos | 41 | East Russia | modern |  | complete mtdna genome | 16774 | [18] |
| 104 | HQ685947 | Ursus | arctos | 47 | West Russia | modern |  | complete mtdna genome | 16774 | [18] |
| 105 | HQ685948 | Ursus | arctos | 48 | West Russia | modern |  | complete mtdna genome | 16785 | [18] |
| 106 | HQ685957 | Ursus | arctos | 57 | North Finland | modern |  | complete mtdna genome | 16774 | [18] |
| 107 | HQ685958 | Ursus | arctos | 58 | South Finland | modern |  | complete mtdna genome | 16782 | [18] |
| 108 | HQ685960 | Ursus | arctos | 60 | North Finland | modern |  | complete mtdna genome | 16770 | [18] |
| 109 | JX196367 | Ursus | arctos | KEN10-UAR100 | Kenai | modern |  | complete mtdna genome | 16412 | [19] |
| 110 | JX196368 | Ursus | arctos | ABC2-Lucky | ABC Islands, Alaska, USA | modern |  | complete mtdna genome | 16412 | [19] |
| 111 | JX196369 | Ursus | arctos | ABC1-051711 | Admiralty Island, Alaska, USA | modern |  | complete mtdna genome | 16409 | [19] |
| 112 | JX196370 | Ursus | maritimus | AK1-542 | Barrow, Alaska, USA | modern |  | complete mtdna genome | 16412 | [19] |
| 113 | JX196371 | Ursus | maritimus | AK2-562 | Diomede, Alaska, USA | modern |  | complete mtdna genome | 16412 | [19] |
| 114 | JX196372 | Ursus | maritimus | AK3-574 | Barrow, Alaska, USA | modern |  | complete mtdna genome | 16412 | [19] |
| 115 | JX196373 | Ursus | maritimus | AK4-2368 | Diomede, Alaska, USA | modern |  | complete mtdna genome | 16412 | [19] |
| 116 | JX196374 | Ursus | maritimus | AK5-651 | Savoonga, Alaska, USA | modern |  | complete mtdna genome | 16412 | [19] |
| 117 | JX196376 | Ursus | maritimus | PB2-N23604 | Svalbard | modern |  | complete mtdna genome | 16412 | [19] |
| 118 | JX196377 | Ursus | maritimus | PB3-N23719 | Svalbard | modern |  | complete mtdna genome | 16412 | [19] |
| 119 | KF182305 | Ursus | arctos | KLE-VI-22 | Southern Europe | unknown |  | partial D-loop | 269 | [20] |
| 120 | KF544785 | Ursus | spelaeus | Altai_D10_51411 | Denisova cave, Russia | ancient | unknown | partial D-loop | 251 | [20] |
| 121 | KF544788 | Ursus | spelaeus | Mt.Generoso_48082 | Mt. Generoso Bear Cave, Italy | ancient | unknown | partial D-loop | 251 | [20] |
| 122 | KF545630 | Ursus | arctos | HK_1UR | Russia | modern |  | control region | 759 | [21] |
| 123 | KF545635 | Ursus | arctos | T_12 | Russia | modern |  | control region | 759 | [21] |
| 124 | KF545637 | Ursus | arctos | hM_E8 | Russia | modern |  | control region | 759 | [21] |
| 125 | KF545639 | Ursus | arctos | U_E49 | Russia | modern |  | control region | 759 | [21] |
| 126 | KF563083 | Ursus | arctos | Y_769 | Russia | modern |  | control region | 570 | [21] |
| 127 | KF563085 | Ursus | arctos | H_337 | Russia | modern |  | control region | 567 | [21] |
| 128 | KM676523 | Ursus | maritimus | 32 | Foxe Basin, Canada | modern |  | partial D-loop | 578 | [22] |
| 129 | KM886408 | Ursus | arctos | SC11 | Sweden | historical (1928) | | partial D-loop | 267 | [23] |
| 130 | KM886414 | Ursus | arctos | SC19 | Norway | historical (1907) | | partial D-loop | 265 | [23] |
| 131 | KM886442 | Ursus | arctos | SC51 | Sweden | historical (1844) | | partial D-loop | 265 | [23] |
| 132 | KP668973 | Ursus | arctos | 1 | Iran | modern |  | partial D-loop | 614 | [24] |
| 133 | KP668974 | Ursus | arctos | 2 | Iran | modern |  | partial D-loop | 614 | [24] |
| 134 | KP668975 | Ursus | arctos | 3 | Iran | modern |  | partial D-loop | 614 | [24] |
| 135 | KP668976 | Ursus | arctos | 4 | Iran | modern |  | partial D-loop | 614 | [24] |
| 136 | KP668977 | Ursus | arctos | 5 | Iran | modern |  | partial D-loop | 614 | [24] |
| 137 | KP668978 | Ursus | arctos | 6 | Iran | modern |  | partial D-loop | 614 | [24] |
| 138 | KP668986 | Ursus | arctos | 14 | Iran | modern |  | partial D-loop | 614 | [24] |
| 139 | KT438621 | Ursus | arctos | TR-1 | Artvin, Turkey | modern |  | partial D-loop | 265 | [25] |
| 140 | KT438634 | Ursus | arctos | TR-21 | Artvin, Turkey | modern |  | partial D-loop | 271 | [25] |
| 141 | KT438636 | Ursus | arctos | TR-23 | Antalya, Turkey | modern |  | partial D-loop | 265 | [25] |
| 142 | KT438637 | Ursus | arctos | TR-24 | Antalya, Turkey | modern |  | partial D-loop | 264 | [25] |
| 143 | KT438639 | Ursus | arctos | TR-26 | Artvin, Turkey | modern |  | partial D-loop | 266 | [25] |
| 144 | KT438640 | Ursus | arctos | TR-27 | Artvin, Turkey | modern |  | partial D-loop | 266 | [25] |
| 145 | KT438651 | Ursus | arctos | TR-Z2 | Turkey | modern |  | partial D-loop | 266 | [25] |
| 146 | KU833227 | Ursus | arctos | Cau11 | Russia | modern |  | partial D-loop | 568 | [26] |
| 147 | KU833228 | Ursus | arctos | Cau10 | Russia | modern |  | partial D-loop | 568 | [26] |
| 148 | KU833229 | Ursus | arctos | Cau9 | Russia | modern |  | partial D-loop | 571 | [26] |
| 149 | KU833232 | Ursus | arctos | CauB6 | Russia | modern |  | partial D-loop | 568 | [26] |
| 150 | KU833238 | Ursus | arctos | CauM6 | Russia | modern |  | partial D-loop | 568 | [26] |
| 151 | KU833239 | Ursus | arctos | AZ2 | Azerbaijan | modern |  | partial D-loop | 568 | [26] |
| 152 | KU833243 | Ursus | arctos | Z1 | Armenia | modern |  | partial D-loop | 568 | [26] |
| 153 | KX641317 | Ursus | arctos | BB_SP4 | Pena Paleira, Lugo, Spain | ancient | 7,452 | complete mtdna genome | 16350 | [27] |
| 154 | KX641331 | Ursus | ingressus | GS136 | Gamssulzen, Austria | ancient | unknown | complete mtdna genome | 16342 | [27] |
| 155 | KY419593 | Ursus | arctos | Arh127 | Russia | modern |  | complete mtdna genome | 16551 | [28] |
| 156 | KY419594 | Ursus | arctos | Arh191 | Russia | modern |  | complete mtdna genome | 16550 | [28] |
| 157 | KY419595 | Ursus | arctos | Est13 | Estonia | modern |  | complete mtdna genome | 16550 | [28] |
| 158 | KY419600 | Ursus | arctos | Fin10_98 | Finland | modern |  | complete mtdna genome | 16550 | [28] |
| 159 | KY419603 | Ursus | arctos | Fin1377 | Finland | modern |  | complete mtdna genome | 16553 | [28] |
| 160 | KY419613 | Ursus | arctos | Hab48 | Russia | modern |  | complete mtdna genome | 16553 | [28] |
| 161 | KY419614 | Ursus | arctos | Hab52 | Russia | modern |  | complete mtdna genome | 16553 | [28] |
| 162 | KY419615 | Ursus | arctos | Kam130 | Russia | modern |  | complete mtdna genome | 16550 | [28] |
| 163 | KY419624 | Ursus | arctos | Kam142 | Russia | modern |  | complete mtdna genome | 16552 | [28] |
| 164 | KY419626 | Ursus | arctos | Kam145 | Russia | modern |  | complete mtdna genome | 16551 | [28] |
| 165 | KY419631 | Ursus | arctos | Kam154 | Russia | modern |  | complete mtdna genome | 16550 | [28] |
| 166 | KY419635 | Ursus | arctos | Kam158 | Russia | modern |  | complete mtdna genome | 16551 | [28] |
| 167 | KY419637 | Ursus | arctos | Kam162 | Russia | modern |  | complete mtdna genome | 16550 | [28] |
| 168 | KY419639 | Ursus | arctos | Kra234 | Russia | modern |  | complete mtdna genome | 16552 | [28] |
| 169 | KY419640 | Ursus | arctos | Kra235 | Russia | modern |  | complete mtdna genome | 16551 | [28] |
| 170 | KY419643 | Ursus | arctos | Len34 | Russia | modern |  | complete mtdna genome | 16550 | [28] |
| 171 | KY419646 | Ursus | arctos | Mag194 | Russia | modern |  | complete mtdna genome | 16550 | [28] |
| 172 | KY419649 | Ursus | arctos | Mag49 | Russia | modern |  | complete mtdna genome | 16549 | [28] |
| 173 | KY419652 | Ursus | arctos | Mag53 | Russia | modern |  | complete mtdna genome | 16554 | [28] |
| 174 | KY419653 | Ursus | arctos | Nov200 | Russia | modern |  | complete mtdna genome | 16551 | [28] |
| 175 | KY419654 | Ursus | arctos | NovS197 | Russia | modern |  | complete mtdna genome | 16550 | [28] |
| 176 | KY419655 | Ursus | arctos | Perm54 | Russia | modern |  | complete mtdna genome | 16551 | [28] |
| 177 | KY419665 | Ursus | arctos | Perm66 | Russia | modern |  | complete mtdna genome | 16552 | [28] |
| 178 | KY419670 | Ursus | arctos | Perm71 | Russia | modern |  | complete mtdna genome | 16550 | [28] |
| 179 | KY419671 | Ursus | arctos | Perm73 | Russia | modern |  | complete mtdna genome | 16551 | [28] |
| 180 | KY419687 | Ursus | arctos | Sver206 | Russia | modern |  | complete mtdna genome | 16554 | [28] |
| 181 | KY419690 | Ursus | arctos | Sver213 | Russia | modern |  | complete mtdna genome | 16551 | [28] |
| 182 | KY419697 | Ursus | arctos | SWE20W | Sweden | modern |  | complete mtdna genome | 16550 | [28] |
| 183 | KY419698 | Ursus | arctos | SWE21E | Sweden | modern |  | complete mtdna genome | 16551 | [28] |
| 184 | KY419702 | Ursus | arctos | Tver203 | Russia | modern |  | complete mtdna genome | 16555 | [28] |
| 185 | MF593957 | Ursus | arctos | ALP1 | Alps, Italy | modern |  | complete mtdna genome | 16750 | [29] |
| 186 | MF593968 | Ursus | arctos | APN12 | Apennine, Italy | modern |  | complete mtdna genome | 16753 | [29] |
| 187 | MG066702 | Ursus | arctos | 70448 | American Museum of Natural History | historical |  | complete mtdna genome | 16388 | [30] |
| 188 | MG066703 | Ursus | arctos | LZHR | Pakistan | modern |  | complete mtdna genome | 16389 | [30] |
| 189 | MG066704 | Ursus | thibetanus | SA | Pakistan | modern |  | complete mtdna genome | 16395 | [30] |
| 190 | MG066705 | Ursus | arctos | YHB | Pakistan | modern |  | complete mtdna genome | 16388 | [30] |
| 191 | MG131889 | Ursus | arctos | TF0017 | Pakistan | modern |  | partial D-loop | 364 | [30] |
| 192 | MG131891 | ursus | arctos | TF0101 | Pakistan | modern |  | partial D-loop | 368 | [30] |

The previously published bear sequences were retrieved from Genbank database with their corresponding accession number, species name, sample ID, sampling location and reference information. The information of sample collection period and age (ancient DNA) were collected from the corresponsing reference papers. The samples <50 years old are modern, 50-300 years old are historical and, >300 years old are considered ancient. The age estimates for ancient DNA were based on uncalibrated radiocarbon (C14) years (years before present, YBP).

References

1. Masuda R, Murata K, Aiurzaniin A, Yoshida MC. Phylogenetic status of brown bears Ursus arctos of Asia: A preliminary result inferred from mitochondrial DNA control region sequences. Hereditas. 1998;128(3):277-80. doi: 10.1111/j.1601-5223.1998.00277.x. PubMed PMID: WOS:000075709500014.

2. Matsuhashi T, Masuda R, Mano T, Murata K, Aiurzaniin A. Phylogenetic relationships among worldwide populations of the brown bear Ursus arctos. Zoological Science. 2001;18(8):1137-43. doi: 10.2108/zsj.18.1137. PubMed PMID: WOS:000173038300012.

3. Delisle I, Strobeck C. Conserved primers for rapid Sequencing of the complete mitochondrial genome from carnivores, applied to three species of bears. Mol Biol Evol. 2002;19(3):357-61. PubMed PMID: WOS:000174127600018.

4. Hofreiter M, Rabeder G, Jaenicke-Despres V, Withalm G, Nagel D, Paunovic M, et al. Evidence for reproductive isolation between cave bear populations. Current Biology. 2004;14(1):40-3. doi: 10.1016/j.cub.2003.12.035. PubMed PMID: WOS:000188118500022.

5. Calvignac S, Hughes S, Tougard C, Michaux J, Thevenot M, Philippe M, et al. Ancient DNA evidence for the loss of a highly divergent brown bear clade during historical times. Mol Ecol. 2008;17(8):1962-70. doi: 10.1111/j.1365-294X.2008.03631.x. PubMed PMID: WOS:000254953900012.

6. Hirata D, Mano T, Abramov AV, Baryshnikov GF, Kosintsev PA, Vorobiev AA, et al. Molecular Phylogeography of the Brown Bear (Ursus arctos) in Northeastern Asia Based on Analyses of Complete Mitochondrial DNA Sequences. Mol Biol Evol. 2013;30(7):1644-52. doi: 10.1093/molbev/mst077. PubMed PMID: WOS:000321056200014.

7. Miller CR, Waits LP, Joyce P. Phylogeography and mitochondrial diversity of extirpated brown bear (Ursus arctos) populations in the contiguous United States and Mexico. Mol Ecol. 2006;15(14):4477-85. doi: 10.1111/j.1365-294X.2006.03097.x. PubMed PMID: WOS:000242042800013.

8. Yu L, Li YW, Ryder OA, Zhang YP. Analysis of complete mitochondrial genome sequences increases phylogenetic resolution of bears (Ursidae), a mammalian family that experienced rapid speciation. Bmc Evol Biol. 2007;7. doi: Artn 19810.1186/1471-2148-7-198. PubMed PMID: WOS:000251904800001.

9. Bon C, Caudy N, de Dieuleveult M, Fosse P, Philippe M, Maksud F, et al. Deciphering the complete mitochondrial genome and phylogeny of the extinct cave bear in the Paleolithic painted cave of Chauvet. P Natl Acad Sci USA. 2008;105(45):17447-52. doi: 10.1073/pnas.0806143105. PubMed PMID: WOS:000260981800052.

10. Korsten M, Ho SYW, Davison J, Pahn B, Vulla E, Roht M, et al. Sudden expansion of a single brown bear maternal lineage across northern continental Eurasia after the last ice age: a general demographic model for mammals? Mol Ecol. 2009;18(9):1963-79. doi: 10.1111/j.1365-294X.2009.04163.x. PubMed PMID: WOS:000265189400014.

11. Jackson JV, Talbot SL, Farley S. Genetic characterization of Kenai brown bears (Ursus arctos): microsatellite and mitochondrial DNA control region variation in brown bears of the Kenai Peninsula, south central Alaska. Can J Zool. 2008;86(7):756-64. doi: 10.1139/Z08-043. PubMed PMID: WOS:000258504900018.

12. Stiller M, Knapp M, Stenzel U, Hofreiter M, Meyer M. Direct multiplex sequencing (DMPS)-a novel method for targeted high-throughput sequencing of ancient and highly degraded DNA. Genome Res. 2009;19(10):1843-8. doi: 10.1101/gr.095760.109. PubMed PMID: WOS:000270389700017.

13. Murtskhvaladze M, Gavashelishvili A, Tarkhnishvili D. Geographic and genetic boundaries of brown bear (Ursus arctos) population in the Caucasus. Mol Ecol. 2010;19(9):1829-41. doi: 10.1111/j.1365-294X.2010.04610.x. PubMed PMID: WOS:000276794300008.

14. Lindqvist C, Schuster SC, Sun YZ, Talbot SL, Qi J, Ratan A, et al. Complete mitochondrial genome of a Pleistocene jawbone unveils the origin of polar bear (vol 107, pg 5053, 2010). P Natl Acad Sci USA. 2010;107(13):6118-. PubMed PMID: WOS:000276159500077.

15. Hailer F, Kutschera VE, Hallstrom BM, Klassert D, Fain SR, Leonard JA, et al. Nuclear Genomic Sequences Reveal that Polar Bears Are an Old and Distinct Bear Lineage. Science. 2012;336(6079):344-7. doi: 10.1126/science.1216424. PubMed PMID: WOS:000302995400045.

16. Bidon T, Janke A, Fain SR, Eiken HG, Hagen SB, Saarma U, et al. Brown and Polar Bear Y Chromosomes Reveal Extensive Male-Biased Gene Flow within Brother Lineages. Mol Biol Evol. 2014;31(6):1353-63. doi: 10.1093/molbev/msu109. PubMed PMID: WOS:000337067400004.

17. Kocijan I, Galov A, Cetkovic H, Kusak J, Gomercic T, Huber D. Genetic diversity of Dinaric brown bears (Ursus arctos) in Croatia with implications for bear conservation in Europe. Mamm Biol. 2011;76(5):615-21. doi: 10.1016/j.mambio.2010.12.003. PubMed PMID: WOS:000295440100012.

18. Keis M, Remm J, Ho SYW, Davison J, Tammeleht E, Tumanov IL, et al. Complete mitochondrial genomes and a novel spatial genetic method reveal cryptic phylogeographical structure and migration patterns among brown bears in north-western Eurasia. Journal of Biogeography. 2013;40(5):915-27. doi: 10.1111/jbi.12043. PubMed PMID: WOS:000317993000010.

19. Miller W, Schuster SC, Welch AJ, Ratan A, Bedoya-Reina OC, Zhao FQ, et al. Polar and brown bear genomes reveal ancient admixture and demographic footprints of past climate change. P Natl Acad Sci USA. 2012;109(36):E2382-E90. doi: 10.1073/pnas.1210506109. PubMed PMID: WOS:000308912600003.

20. Baca M, Mackiewicz P, Stankovic A, Popovic D, Stefaniak K, Czarnogorska K, et al. Ancient DNA and dating of cave bear remains from Niedzwiedzia Cave suggest early appearance of Ursus ingressus in Sudetes. Quatern Int. 2014;339:217-23. doi: 10.1016/j.quaint.2013.08.033. PubMed PMID: WOS:000340328200020.

21. Salomashkina VV, Kholodova MV, Tuten'kov OY, Moskvitina NS, Erokhin NG. New data on the phylogeography and genetic diversity of the brown bear Ursus arctos Linnaeus, 1758 of Northeastern Eurasia (mtDNA control region polymorphism analysis). Biology Bulletin. 2014;41(1):38-46. doi: 10.1134/S1062359014010087. PubMed PMID: WOS:000330775300006.

22. Peacock E, Sonsthagen SA, Obbard ME, Boltunov A, Regehr EV, Ovsyanikov N, et al. Implications of the Circumpolar Genetic Structure of Polar Bears for Their Conservation in a Rapidly Warming Arctic. Plos One. 2015;10(1). doi: ARTN e11202110.1371/journal.pone.0112021. PubMed PMID: WOS:000348160000001.

23. Xenikoudakis G, Ersmark E, Tison JL, Waits L, Kindberg J, Swenson JE, et al. Consequences of a demographic bottleneck on geneticstructure and variation in the Scandinavian brown bear. Mol Ecol. 2015;24(13):3441-54. doi: 10.1111/mec.13239. PubMed PMID: WOS:000356973100018.

24. Ashrafzadeh MR, Kaboli M, Naghavi MR. Mitochondrial DNA analysis of Iranian brown bears (Ursus arctos) reveals new phylogeographic lineage. Mamm Biol. 2016;81(1):1-9. doi: 10.1016/j.mambio.2015.09.001. PubMed PMID: WOS:000369462600001.

25. Cilingir FG, Peksen CA, Ambarli H, Beerli P, Bilgin CC. Exceptional maternal lineage diversity in brown bears (Ursus arctos) from Turkey. Zool J Linn Soc-Lond. 2016;176(2):463-77. doi: 10.1111/zoj.12322. PubMed PMID: WOS:000368930100008.

26. Salomashkina VV, Kholodova MV, Semenov UA, Muradov AS, Malkhasyan A. Genetic variability of brown bear (Ursus arctos L., 1758). Russ J Genet+. 2017;53(1):108-17. doi: 10.1134/S1022795416120103. PubMed PMID: WOS:000394159700011.

27. Fortes GG, Grandal-d'Anglade A, Kolbe B, Fernandes D, Meleg IN, Garcia-Vazquez A, et al. Ancient DNA reveals differences in behaviour and sociality between brown bears and extinct cave bears. Mol Ecol. 2016;25(19):4907-18. doi: 10.1111/mec.13800. PubMed PMID: WOS:000384810000016.

28. Anijalg P, Ho SYW, Davison J, Keis M, Tammeleht E, Bobowik K, et al. Large-scale migrations of brown bears in Eurasia and to North America during the Late Pleistocene. Journal of Biogeography. 2018;45(2):394-405. doi: 10.1111/jbi.13126. PubMed PMID: WOS:000424642900011.

29. Benazzo A, Trucchi E, Cahill JA, Delser PM, Mona S, Fumagalli M, et al. Survival and divergence in a small group: The extraordinary genomic history of the endangered Apennine brown bear stragglers. P Natl Acad Sci USA. 2017;114(45):E9589-E97. doi: 10.1073/pnas.1707279114. PubMed PMID: WOS:000414631200021.

30. Lan TY, Gill S, Bellemain E, Bischof R, Nawaz MA, Lindqvist C. Evolutionary history of enigmatic bears in the Tibetan Plateau - Himalaya region and the identity of the yeti. P Roy Soc B-Biol Sci. 2017;284(1868). doi: ARTN 2017180410.1098/rspb.2017.1804. PubMed PMID: WOS:000417194500015.
