# Supplementary material for: Phylogeography, genetic diversity, and connectivity of brown bear populations in Central Asia
Source: PLoS One. 2019 Aug 13;14(8):e0220746. doi: 10.1371/journal.pone.0220746 (PMC6692007; doi:10.1371/journal.pone.0220746)
Supplement: S2 Table — The concatenated sequencing dataset includes 671 bp COXII and 261bp Control Region. Twenty-two new haplotypes for control region sequences and 12 new haplotypes from COXII were identified in this study. Concatenated column gives the sample ID used in the phylogenetic and divergence time analyses. The previously reported haplotypes are indicated with the symbol (§) and the Genbank ID is in parentheses in the sample ID column. (DOCX) [file pone.0220746.s002.docx]

**S2 Table. Brown bear haplotype distribution in Central Asia based on mitochondrial DNA sequence data**

| Location  (Approximate GPS location) | Clade | Concatenated (927bp) | Control Region Haplotypes (256bp) | Number of samples | Sample names | Clade | COXII Haplotypes (671bp) | Number of samples | Sample names |
| --- | --- | --- | --- | --- | --- | --- | --- | --- | --- |
| Gobi  (~43°40´N; ~97°20´E) | 6 | 1001 | CR_Hap1 | 13 | 1001, 10014, 1002, 1003Kho, 1006, 1007, 1009, 100G4, 100G5, 100G6, 100Gobi11, 100Gobi12, 100Gobi2, (AB010728 [1]) | 6 | COII_Hap1 | 14 | 1001, 10014, 1002, 1005, 1006, 1007, 1008, 1009, 100G4, 100G5, HGB7, HGB8, HGb9, 100S1 |
|  |  |  | CR_Hap2 | 1 | 100G3 |  |  |  |  |
| Altai  (~47°52´N; ~90°88´E) | 3b1 | 20023BU | CR_Hap3 | 1 | 20023BU | 3b1 | COII_Hap2 | 7 | 20023BU, 20027BU, 20028BU, 200BUS1, 200S5, 200S7, 200S8 |
|  | 3b1 | 200BU27 | CR_Hap4 | 5 | 20027BU, 20028BU, 200BU4, 200BU5, 200BU6 |  |  |  |  |
|  | 3b1 | 200BU1 | CR_Hap5 | 1 | 200BU1 | 3b1 | COII_Hap3 | 3 | 200BU1, 200S10, 200S4 |
|  | 3b1 | 200BU3 | CR_Hap6 | 1 | 200BU3 | 3b1 | COII_Hap4 | 1 | 200BU3 |
|  | 3b1 |  | CR_Hap7 | 1 | 200S4 |  |  |  |  |
| Khentii  (~48°43´N; ~108°30´E) | 3a1 | 30036Khe | CR_Hap8 | 8 | 30036Khe, 30040Khe, 30041Khe, 300Br40Khe, 300Khe1, 300Khe3, 300Khe9, 300S35 | 3a1 | COII_Hap4 | 5 | 30036Khe, 30040Khe, 30041Khe, 300S22 |
|  |  | 300Br41Khe |  |  |  |  |  |  |  |
|  | 3b2 | 30037Khe | CR_Hap9 | 1 | 30037Khe | 3b2 | COII_Hap5 | 4 | 300S25, 300S27, 300S28, 300S29, 300S33 |
|  | 3b2 | 30038Khe | CR_Hap10 | 1 | 30038Khe | 3b2 | COII_Hap6 | 7 | 30037Khe, 30038Khe, 30039Khe, 300S23, 300S26, 300S35, 300S36 |
|  | 3b2 | 300Br42Khe | CR_Hap12 | 2 | 300Br42Khe, 300Khe10 | 3b2 | COII_Hap7 | 1 | 300S31 |
|  | 3b2 | 300S23 | CR_Hap13 | 12 | 300Br43Khe, 300Khe11, 300Khe12, 300Khe13, 300Khe14, 300Khe4, 300Khe6, 300Khe7, 300Khe8, 300S23, 300S25, 300S33 |  |  |  |  |
|  | 3b2 | 300S27 | CR_Hap14 | 1 | 300S27 |  |  |  |  |
|  | 3b2 |  | CR_Hap15 | 1 | 300S36 |  |  |  |  |
| Buteeliin Nuruu  (~49°59´N; ~104°07´E) | 3a1 | 4004Sel | CR_Hap8 | 2 | 4004Sel, 4006Sel (EF033738 [2]) | 3a1 | COII_Hap5 | 3 | 4001Sel, 4004Sel, 4005Sel (AP012579 [3]; JX196367 [4], KY419639-40, KY419646-51, KY419654, KY419674, KY419687, KY419692-94 [5]) |
|  | 3b2 | 4004S13 |  |  |  |  |  |  |  |
|  | 3b2 | 40010Sel | CR_Hap13 | 8 | 40010Sel, 400S13, 400S14, 400Se2, 400Se3, 400Se4, 400Se6, 400Se8 | 3b2 | COII_Hap4 | 3 | 400S13, 400S14, 400S15 |
|  |  | 400Se6 |  |  |  |  |  |  |  |
|  | 3b2 | 4008Sel | CR_Hap15 | 1 | 4008Sel | 3b2 | COII_Hap6 | 2 | 40010Sel, 400S21 |
|  | 3a1 | 400Se5 | CR_Hap16 | 1 | 400Se5 (EF033845 [2], AB041258 [6]) | 3b2 | COII_Hap8 | 1 | 4008Sel |
|  |  |  |  |  |  | 3b2 | COII_Hap9 | 1 | 4009Sel |
| Bogd Khan  (~47°40´N; ~107°10´E) | 3b1 | 70032Tov | CR_Hap18 | 1 | 32Tov | 3a1 | COII_Hap5 | 1 | 32Tov |
|  | 3b2 | 70033Tov | CR_Hap24 | 1 | 33Tov | 3b2 | COII_Hap11 | 1 | 33Tov |
|  | 3a1 | 70035Tov | CR_Hap25 | 1 | 35Tov | 3a1 | COII_Hap12 | 1 | 35Tov |
| Sayan  (~51°20´N; ~101°15´E) | 3b2 | 50031Khu | CR_Hap13 | 3 | 50030Khu, 50031Khu, 500S2 | 3b2 | COII_Hap4 | 2 | 500Khu2, 50031Khu |
|  | 3b2 | 50030Khu | CR_Hap17 | 1 | 50025Khu | 3b2 | COII_Hap10 | 3 | 50025Khu, 50030Khu, 500S2 |
|  | 3b2 | 50025Khu | CR_Hap18 | 1 | 500Br44Khu |  |  |  |  |
|  | 3a1 | 500Br45Khu | CR_Hap19 | 1 | 500Br45Khu |  |  |  |  |
|  | 3b2 |  | CR_Hap20 | 1 | 500Khu2 |  |  |  |  |
|  | 3b2 |  | CR_Hap23 | 1 | 500Khu1 |  |  |  |  |
| Ikh Khyangan  (~46°90´N; ~119°20´E) | 3b2 | 60020Dor | CR_Hap18 | 1 | 60020Dor | 3b2 | COII_Hap4 | 3 | 60014Dor, 60018Dor, 60019Dor |
|  | 3b3 | 60014Dor | CR_Hap20 | 1 | 60014Dor |  |  |  |  |
|  | 3b3 | 60017Dor | CR_Hap21 | 1 | 60017Dor |  |  |  |  |
|  | 3b2 | 60019Dor | CR_Hap22 | 1 | 60019Dor |  |  |  |  |
| Himalaya  (~35°50´N; ~75°20´E) | 6 | 800Pak1 | CR_Hap26 | 3 | 800Pak1, Pak4, Pak5, (DQ914409 [7]) | 6 | Hap13 | 5 | 800Pak1, Pak2, Pak3, Pak4, Pak5 |
| Total |  | 31 | 26 | 79 |  |  | 13 | 68 |  |

The concatenated sequencing dataset includes 671 bp COXII and 256 bp Control Region. Twenty-two new haplotypes for control region sequences and 12 new haplotypes from COXII were identified in this study. Concatenated column gives the sample ID used in the phylogenetic and divergence time analyses.

References

1. Masuda R, Murata K, Aiurzaniin A, Yoshida MC. Phylogenetic status of brown bears Ursus arctos of Asia: A preliminary result inferred from mitochondrial DNA control region sequences. Hereditas. 1998;128(3):277-80. doi: 10.1111/j.1601-5223.1998.00277.x. PubMed PMID: WOS:000075709500014.

2. Waits LP, Talbot SL, Ward RH, Shields GF. Mitochondrial DNA phylogeography of the North American brown bear and implications for conservation. Conservation Biology. 1998;12(2):408-17. doi: 10.1046/j.1523-1739.1998.96351.x. PubMed PMID: WOS:000073215200019.

3. Hirata D, Mano T, Abramov AV, Baryshnikov GF, Kosintsev PA, Vorobiev AA, et al. Molecular Phylogeography of the Brown Bear (Ursus arctos) in Northeastern Asia Based on Analyses of Complete Mitochondrial DNA Sequences. Mol Biol Evol. 2013;30(7):1644-52. doi: 10.1093/molbev/mst077. PubMed PMID: WOS:000321056200014.

4. Miller W, Schuster SC, Welch AJ, Ratan A, Bedoya-Reina OC, Zhao FQ, et al. Polar and brown bear genomes reveal ancient admixture and demographic footprints of past climate change. P Natl Acad Sci USA. 2012;109(36):E2382-E90. doi: 10.1073/pnas.1210506109. PubMed PMID: WOS:000308912600003.

5. Anijalg P, Ho SYW, Davison J, Keis M, Tammeleht E, Bobowik K, et al. Large-scale migrations of brown bears in Eurasia and to North America during the Late Pleistocene. Journal of Biogeography. 2018;45(2):394-405. doi: 10.1111/jbi.13126. PubMed PMID: WOS:000424642900011.

6. Matsuhashi T, Masuda R, Mano T, Murata K, Aiurzaniin A. Phylogenetic relationships among worldwide populations of the brown bear Ursus arctos. Zoological Science. 2001;18(8):1137-43. doi: 10.2108/zsj.18.1137. PubMed PMID: WOS:000173038300012.

7. Miller CR, Waits LP, Joyce P. Phylogeography and mitochondrial diversity of extirpated brown bear (Ursus arctos) populations in the contiguous United States and Mexico. Mol Ecol. 2006;15(14):4477-85. doi: 10.1111/j.1365-294X.2006.03097.x. PubMed PMID: WOS:000242042800013.
