# Supplementary material for: Phylogeography, genetic diversity, and connectivity of brown bear populations in Central Asia
Source: PLoS One. 2019 Aug 13;14(8):e0220746. doi: 10.1371/journal.pone.0220746 (PMC6692007; doi:10.1371/journal.pone.0220746)
Supplement: S3 Table — Description: n, number of individuals; S, variable sites; Haplotype (gene) diversity, Hd; Nucleotide diversity, π; Average number of nucleotide differences, K. (DOCX) [file pone.0220746.s003.docx]

**S3 Table. Genetic diversity estimates within-population based on brown bear COXII mitochondrial DNA data (671 bp).**

| Sampling Location | N | Number of Haplotypes | S | Hd | π | K |
| --- | --- | --- | --- | --- | --- | --- |
| Gobi (GGSPA Mongolia) | 14 | 1 | 0 | 0.0000 | 0.0000 | 0.0000 |
| Khentii (Khentii, Buteeliin nuruu and Bogd Khan) | 27 | 8 | 9 | 0.8006 | 0.0051 | 3.3846 |
| Sayan (Khuvsgul) | 5 | 2 | 1 | 0.6000 | 0.0009 | 0.6000 |
| Altai | 11 | 3 | 3 | 0.5636 | 0.0021 | 1.3818 |
| (Bayan-Ulgii) |  |  |  |  |  |  |
| Khingan (Dornod) | 3 | 1 | 0 | 0.0000 | 0.0000 | 0.0000 |
| Himalaya (Pakistan) | 5 | 1 | 1 | 0.0000 | 0.0000 | 0.0000 |
| Total | 65 | 13 | 23 | 0.8774 | 0.0105 | 7.0308 |

n, number of individuals; S, variable sites; Haplotype (gene) diversity, Hd; Nucleotide diversity, π ; Average number of nucleotide differences, K
