# Supplementary material for: Phylogeography, genetic diversity, and connectivity of brown bear populations in Central Asia
Source: PLoS One. 2019 Aug 13;14(8):e0220746. doi: 10.1371/journal.pone.0220746 (PMC6692007; doi:10.1371/journal.pone.0220746)
Supplement: S4 Table — Description: Gaps are excluded and the total sites 243 bp out of 261 bp. n, number of individuals; S, variable sites; Haplotype (gene) diversity, Hd; Nucleotide diversity, π; Average number of nucleotide differences, K. (DOCX) [file pone.0220746.s004.docx]

**S4 Table. Genetic diversity within populations, based on brown bear Control Region mitochondrial DNA data**

| Sampling Location | n | Number of Haplotypes | S | Hd | π | K |
| --- | --- | --- | --- | --- | --- | --- |
| Gobi Desert (GGSPA) | 14 | 2 | 6 | 0.14286 | 0.00351 | 0.85714 |
| Khentii (Khentii, Buteeliin nuruu and Bogd Khan) | 37 | 13 | 11 | 0.77928 | 0.01067 | 2.6036 |
| Sayan (Khuvsgul) | 8 | 6 | 9 | 0.89286 | 0.01273 | 3.10714 |
| Altai (Bayan-Ulgii) | 9 | 5 | 8 | 0.72222 | 0.00797 | 1.94444 |
| Khingan (Dornod) | 4 | 4 | 11 | 1 | 0.02527 | 6.16667 |
| Himalaya (Pakistan) | 3 | 2 | 1 | 0.4 | 0.00165 | 0.4 |
| Total | 79 | 25 | 31 | 0.89261 | 0.02719 | 6.63423 |

Gaps are excluded and the total sites 243 bp out of 261 bp. n, number of individuals; S, variable sites; Haplotype (gene) diversity, Hd; Nucleotide diversity, π ; Average number of nucleotide differences, K
