# Supplementary figures and images for: Phylogeography, genetic diversity, and connectivity of brown bear populations in Central Asia
Source: PLoS One. 2019 Aug 13;14(8):e0220746. doi: 10.1371/journal.pone.0220746 (PMC6692007; doi:10.1371/journal.pone.0220746)

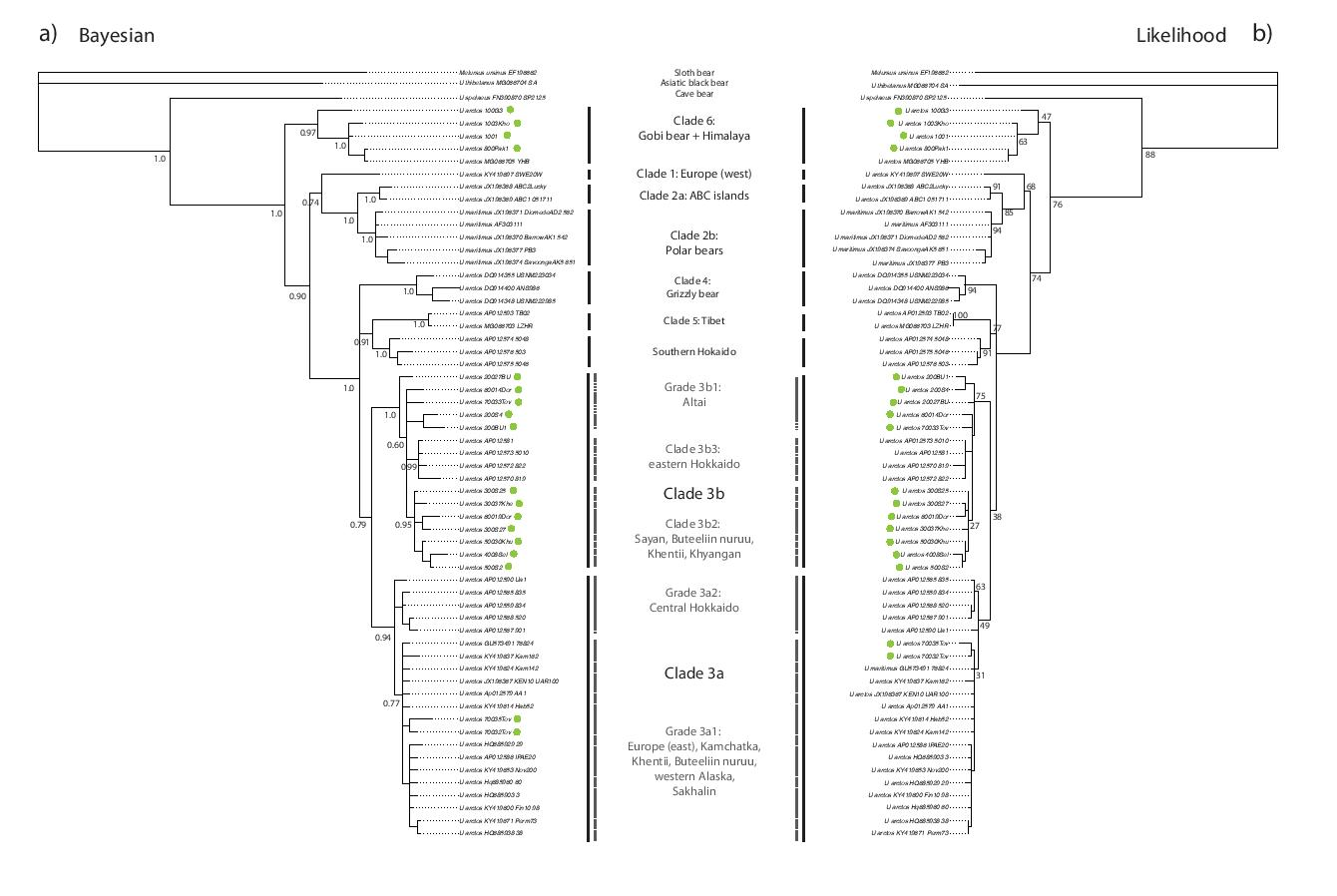

Supplement: S1 Fig — The concatenated sequencing dataset (927 bp) includes 671 bp COXII and 256 bp Control Region. a) Bayesian and b) Likelihood analyses. The samples with green dots are from our study. (TIF) [file pone.0220746.s005.tif]

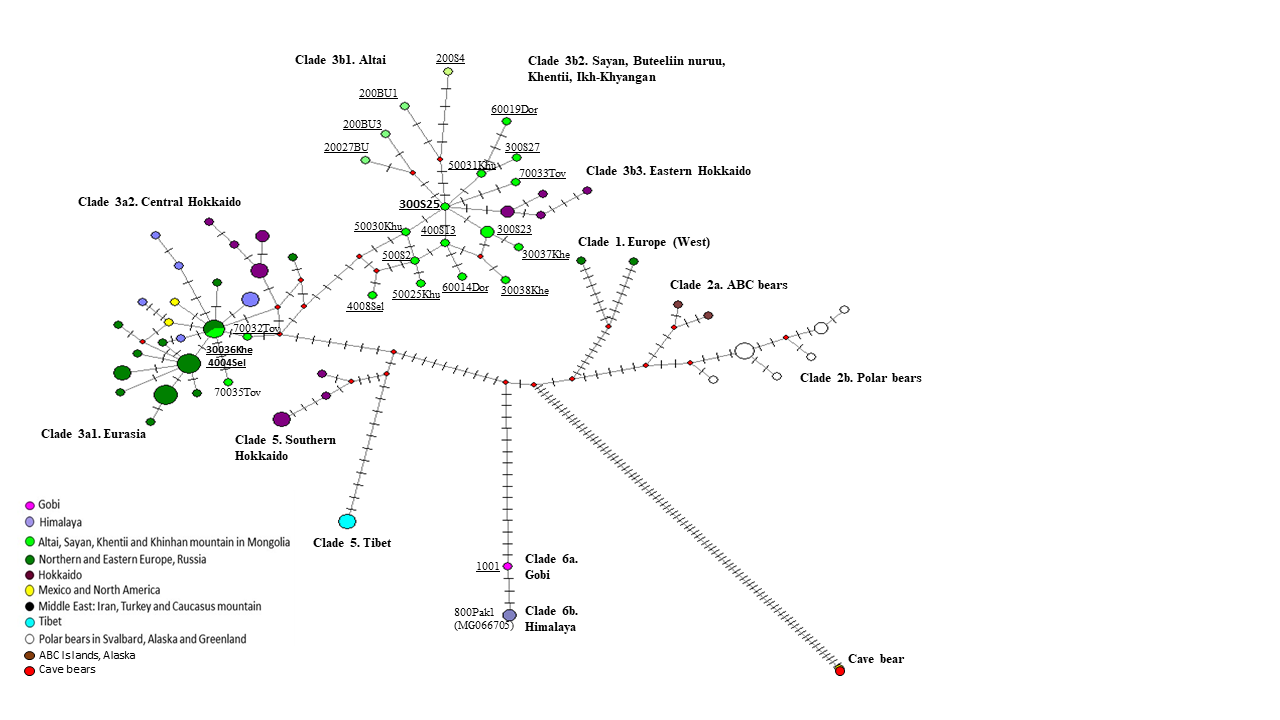

Supplement: S2 Fig — The concatenated sequencing dataset (927 bp) includes 671 bp COXII and 256 bp Control Region. Colors in each node indicate the geographic sampling locations; the size of the node indicate frequency of the haplotype. The hatches represent mutational differences in the sequences. The names of the new haplotypes discovered in this study are given in the underlined text on the nodes. (TIF) [file pone.0220746.s006.tif]

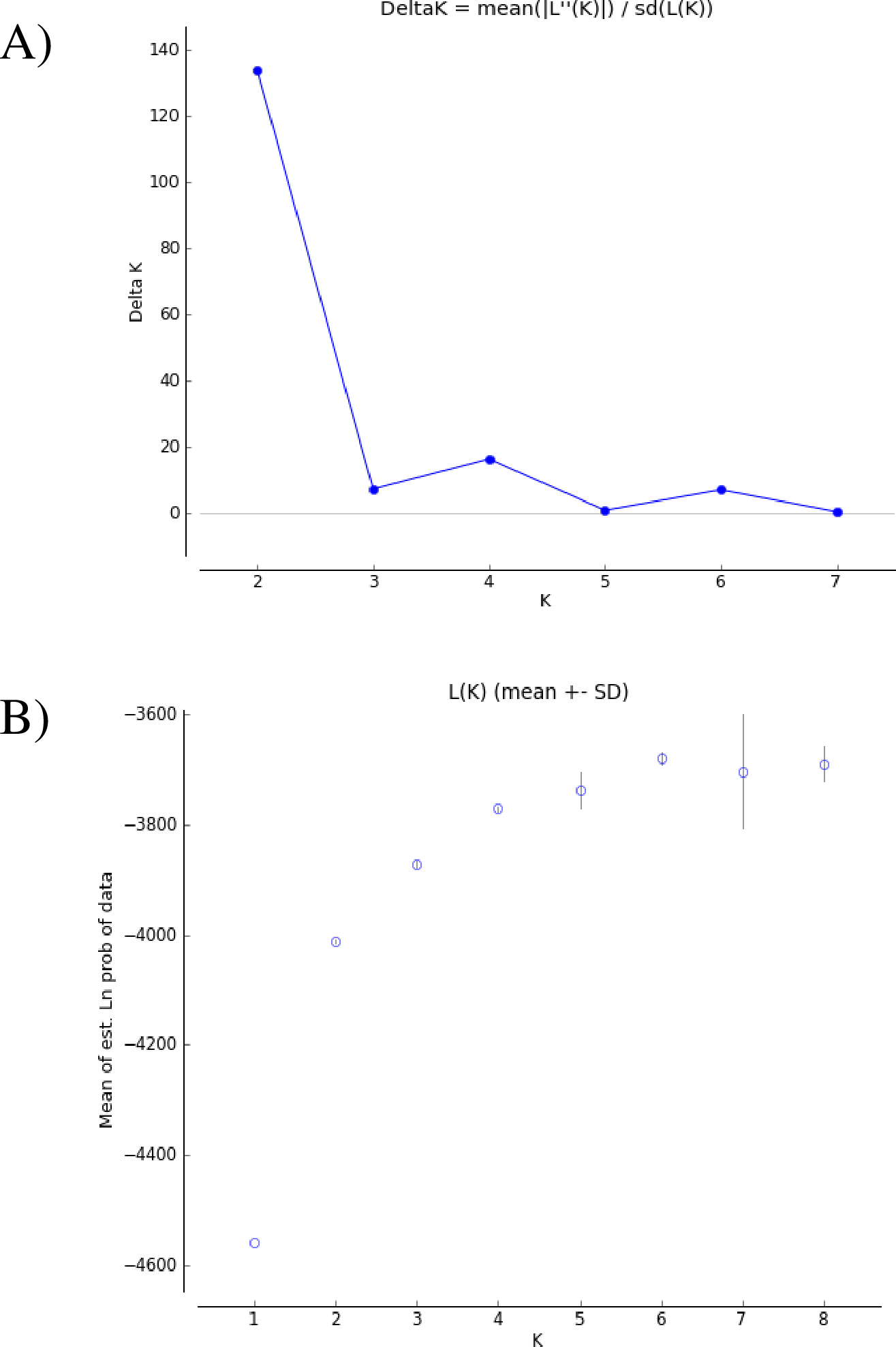

Supplement: S3 Fig — The results are based on 13 loci microsatellite dataset of Asian brown bears. A) The DeltaK results support K = 2 as the most basic level of subdivision (Fig 4), which splits the Gobi bears from the Himalayan-Eurasian Brown bears in the STRUCTURE analysis. B) STRUCTURE likelihood curve for K = 1–8. (TIF) [file pone.0220746.s007.tif]

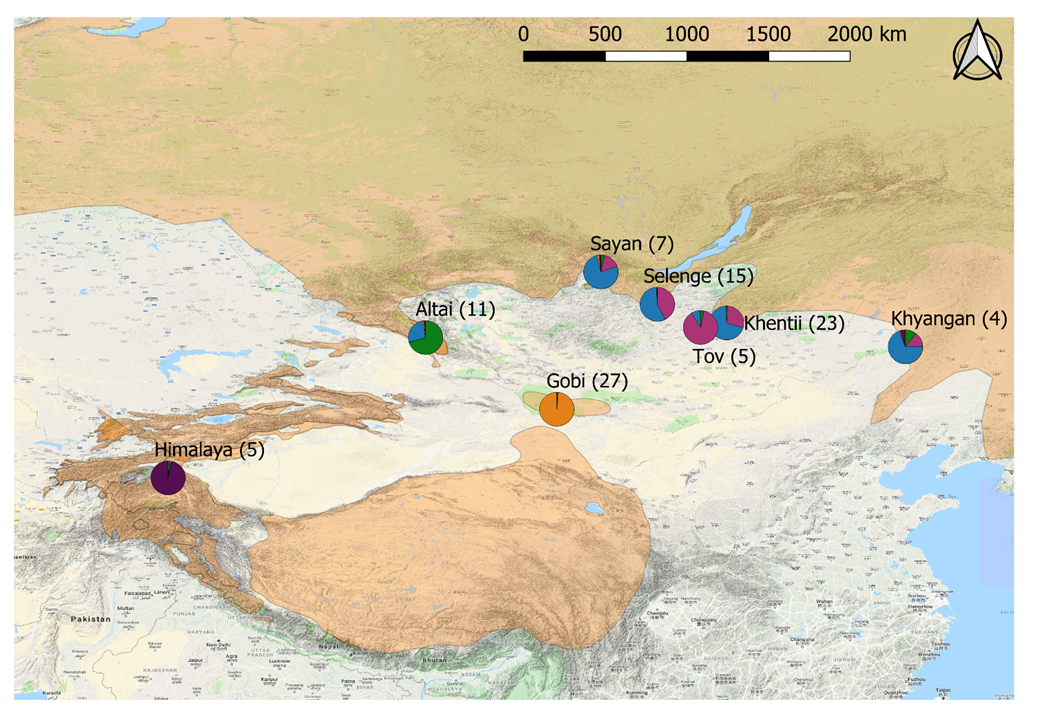

Supplement: S4 Fig — Colors and pie charts represent proportions of the ancestry belonging to each K. Pie charts and names (with sample size) are in the general locations. The current brown bear distribution [6] is highlighted as orange. (TIF) [file pone.0220746.s008.tif]
